# Optimizing skin sensitization prediction across activity cliffs: a comparative analysis of K-nearest neighbours vs random Forest

**DOI:** 10.3389/ftox.2026.1739746

**Published:** 2026-08-03

**Authors:** Daniel C. Ukaegbu, Karolina Kopańska, Peter Ranslow, Alexandra Maertens

**Affiliations:** 1 Center for Alternatives to Animal Testing (CAAT), Johns Hopkins Bloomberg School of Public Health, Baltimore, MD, United States; 2 Consortium for Environmental Risk Management (CERM), Hallowell, ME, United States

**Keywords:** chemical similarity maps, machine learning, molecular fingerprint, skin sensitization, structural alert

## Abstract

Computational models for skin sensitization prediction face critical challenges in handling activity cliffs, where structurally similar compounds exhibit different biological activities, limiting their regulatory applicability. This study systematically compared Random Forest (RF) and K‐Nearest Neighbors (KNN) models using five molecular fingerprint approaches integrated with structural alerts and physicochemical properties. Models were developed using 1174 chemicals and evaluated across progressive feature integration levels: fingerprints alone, fingerprints with structural alerts, and fully integrated models. Performance was assessed using standard classification metrics and chemical similarity analysis for compounds with ≥70% Tanimoto similarity but discordant experimental outcomes. RF consistently outperformed KNN across all fingerprint approaches, achieving 81% balanced accuracy compared to (74%) in fully integrated models on the test set. Critically, RF demonstrated superior handling of activity cliffs compared to KNN. Substructure‐based fingerprints (Avalon, PubChem and MACCS) consistently outperformed hash‐based approaches (Morgan, Atom Pair), with Avalon showing optimal performance across metrics. SHAP interpretability analysis identified vapor pressure (VP) as the most consistently important physicochemical predictor and identified key reactive structural features aligning with known sensitization mechanisms. These findings provide evidence‐based guidance for model selection when developing tools for skin sensitization and establish a systematic methodology for evaluating activity cliff performance that can be applied across other toxicological endpoints.

## Introduction

Skin sensitization, or allergic contact dermatitis (ACD), is a common occupational hazard with significant implications for public health and chemical safety regulation. It accounts for a substantial proportion of work-related skin diseases and can lead to chronic conditions, reduced quality of life, and economic burden due to lost productivity and medical costs (Adamson, 2024; Kalboussi et al., 2019). Mechanistically, skin sensitization is triggered when an individual is exposed to an electrophilic substance that binds to skin proteins, initiating the production of allergen-specific T cells during the induction or sensitization phase. Upon re-exposure, these primed T cells trigger the elicitation phase, completing the adverse outcome pathway (AOP) for skin sensitization (OECD, 2012).

For many years, the Local Lymph Node Assay (LLNA) has served as the regulatory gold standard for evaluating the skin sensitization potential of chemicals, particularly within the context of the REACH Regulation and OECD Test Guideline 429 (OECD, 2010). Alongside earlier *in vivo* methods such as the Guinea Pig Maximization Test (Magnusson and Kligman, 1969) and the Buehler Test (OECD, 1992), the LLNA gained widespread adoption due to its ability to quantitatively assess allergic contact dermatitis risk (Gerberick and Robinson, 2000; Williams et al., 2015).

However, *in vivo* assays like the LLNA have faced increasing criticism due to their high cost, ethical concerns, and their translation to humans (Luechtefeld et al., 2016). These limitations have catalyzed a shift toward non-animal testing strategies that aim to improve both mechanistic understanding and human relevance in predictive toxicology. In response, a suite of validated *in vitro* assays has been developed to investigate key biological events in the skin sensitization Adverse Outcome Pathway (OECD, 2014). These include the Direct Peptide Reactivity Assay (DPRA), which evaluates a chemical’s ability to form covalent bonds with nucleophilic residues (Gerberick et al., 2004); the Keratinases™ assay, which measures the activation of keratinocytes via antioxidant response element (ARE)-driven luciferase expression (Ramirez et al., 2014); and the Human Cell Line Activation Test (h-CLAT), which assesses dendritic cell activation markers such as CD86 and CD54 (Alépée et al., 2015; Ashikaga et al., 2006). These and other methods have been recommended by EURL ECVAM and are incorporated in guidance documents by the OECD (OECD, 2016).

Collectively, these assays have been integrated into Defined Approaches (DAs) under the broader framework of Integrated Approaches to Testing and Assessment (IATA) to support weight-of-evidence decision-making in chemical risk assessment (Kleinstreuer et al., 2018).

While this represents significant progress, these approaches are not without limitations. Critically, each *in vitro* method addresses only a single key event in the AOP, and even integrated strategies cannot fully reproduce the complex physiological context of an *in vivo* immune response (Sewell et al., 2024). As a result, no single method or even a battery of them can yet provide a complete and consistent prediction of sensitization potency across the diversity of chemical structures encountered in real-world applications (Wareing et al., 2024). This reveals a central challenge in the implementation of New Approach Methodologies (NAMs): the integration of mechanistic, *in silico*, and empirical data into coherent and reliable frameworks suitable for regulatory decision-making (Gilmour et al., 2019).

Despite the development of various alternative approaches, a standalone test for skin sensitization assessment remains elusive (Natsch, 2013). Individual methods, even integrated ones, have inherent limitations. The complex physiological and biochemical mechanisms underlying skin sensitization further suggest that no single approach can adequately predict a chemical’s sensitization potency (Rovida et al., 2015; Urbisch et al., 2015). Many existing methods are confined to laboratory settings and do not provide rapid results, hindering their utility for regulatory purposes.

Aside from the *in vitro* and *in vivo* approaches, numerous computational or *in silico* tools exist for skin sensitization hazard assessment (Alves et al., 2018; Wilm et al., 2018). They employ classification-based techniques, using structure-activity relationships that have been computationally modeled to predict the likelihood that a chemical will cause skin sensitization (Chaudhry et al., 2010; Hartung, 2008; Luechtefeld and Hartung, 2017).

While machine learning offers the potential for timely predictions, these computational approaches often suffer from suboptimal performance, including over- and under-prediction. These limitations frequently arise from chemical “blind spots” that fall outside the applicability domain as well as activity cliffs (Golden et al., 2023).

Studies have demonstrated that computational tools for skin sensitization prediction can achieve accuracy of approximately 70%–80% or higher when validated against human data (Golden et al., 2021). However, this performance is highly dependent on several factors, including the quality and volume of the data, the level of data integration (e.g., combining *in vitro* datasets with molecular descriptors), and the choice of model architecture. The level and type of data integration play a particularly significant role in influencing model performance. Models that integrate diverse data sources such as *in vitro* assay results, physicochemical properties, structural alerts, reactivity profiles, and molecular descriptors can enhance predictive performance (Sarath Kumar et al., 2016). Among these descriptors, molecular fingerprints represent a key subclass that encodes structural features, substructures, or connectivity patterns in binary or count-based formats. Given the diverse types of molecular descriptors (including topological, physicochemical, quantum mechanical, and fragment-based), fingerprints serve as a powerful tool for capturing chemical similarity and pattern recognition essential for machine learning applications. This is because they can capture intricate patterns and relationships underlying skin sensitization more effectively (Golden et al., 2023; Natsch and Gerberick, 2022). By integrating diverse data sources such as *in vitro* assays, physicochemical properties, structural alerts, and molecular descriptors, models can develop a more comprehensive understanding of the mechanisms driving sensitization, improving their generalizability across structurally diverse compounds (Zang et al., 2017). However, this increased data diversity can also introduce challenges. Greater heterogeneity may raise computational costs and lead to model inefficiencies, particularly when algorithms are not well-suited to integrating these data. In such cases, rather than enhancing performance, the additional complexity may reduce a model’s ability to learn relevant patterns or generalize effectively to the underlying mechanisms they were trained in, leading to reduced predictive performance. These challenges point to the importance of balancing data diversity with model complexity and computational feasibility to ensure robust and reliable predictions (Seal et al., 2025).

While *in silico* approaches have demonstrated sub-optimal performance in some instances, particularly when dealing with structurally similar compounds that exhibit contradictory biological activities, they have been vital to advancing skin sensitization prediction (Ta et al., 2021).

However, computational models for skin sensitization stand to benefit from several key advances: systematic evaluation of different machine learning algorithms and molecular descriptors, enhanced understanding of model performance in chemically challenging regions, and improved handling of activity cliffs where small structural changes lead to significant differences in sensitization potential. These challenges point to the importance of understanding how different models handle activity cliffs and how data integration may influence predictive performance in these difficult cases (Golden et al., 2023). These represent critical knowledge gaps, as they pose major challenges for predictive models and are often under-investigated in skin sensitization hazard assessment.

To investigate this problem, we employed RF and KNN models, two widely used machine learning approaches successfully applied to *in silico* skin sensitization prediction (Borba et al., 2021; Koutroumpa et al., 2025; Scheufen Tieghi et al., 2024; Wilm et al., 2018).

Our study evaluates the performance of these prominent machine learning models, using five fingerprint approaches integrated with structural alerts and physicochemical properties. A key focus of our investigation was performance based on chemical similarity: an area often under-investigated in the context of activity cliffs for skin sensitization hazard assessment. By incorporating chemical similarity analysis, we aimed to assess how well these models handle such complexities and predict sensitization status for structurally similar compounds with discordant experimental outcomes. This approach offers valuable insights into model behavior in chemically challenging regions, providing a deeper understanding of the strengths and limitations of these predictive strategies for skin sensitization.

Our research makes three key contributions: (1) demonstrates that ensemble methods (RF) outperform instance-based approaches (KNN) for activity cliff prediction in skin sensitization especially where feature integration is important; (2) shows that substructure-based molecular fingerprints may be more effective than hash-based approaches for this specific endpoint; and (3) provides an interpretability framework using SHAP analysis that identifies both reactive structural features and physicochemical modulators of sensitization potential.

These findings have direct implications for regulatory toxicology, providing guidance for model selection and validation approaches that support the transition from animal testing to computational risk assessment.

## Methods

### Dataset

Initial data curation involved querying e-Chemportal (https://www.echemportal.org/echemportal/substance-search) for skin-sensitizing chemicals, yielding 2626 chemicals. After removing entries with invalid CAS registry numbers (CASRNs), InChI keys were obtained via PubChem’s Python library. The European Chemicals Agency (ECHA) dossier database provided toxicological information, searched based on Classification, Labelling and Packaging (CLP) and skin sensitization experimental results, manually checking for experimental confirmation (with overall a high level of concordance between the two). The data curation process retained 827 sensitizers and 164 non-sensitizers with valid sensitization status documentation. To address this imbalance, 333 negative chemicals were incorporated from (Golden et al., 2021), followed by 164 chemicals from the USEPA high-production volume (HPVC) chemical database (https://cdxapps.epa.gov/oms-substance-registry-services/substance-list-details/74). From these, we could confirm 134 as non-sensitizers based on their ECHA dossier; 30 had no information on their sensitization status and were treated as provisional negatives, bringing the total to 1458 chemicals. The Simplified Molecular Input Line Entry System (SMILES) strings were obtained through PubChem REST API or manually from PubChem using InChI keys. After excluding metals and metal salts as they do not necessarily follow the AOP that traditional organic chemicals follow and thus are outside the scope of this assessment, the final dataset contained 1174 chemicals suitable for model training and testing (518 non-sensitizer compounds and 656 sensitizers).

### Descriptors

#### Fingerprint

Molecular fingerprints were generated via RDKit cheminformatics package version 2022.09.5, except for PubChem fingerprint obtained through the PubChem [https://ftp.ncbi.nlm.nih.gov/pubchem/specifications/pubchem_fingerprints.pdf]. Five fingerprint types were employed: MACCS (166 bits), utilizing predefined SMARTS patterns (Kwon et al., 2021); Avalon (1024 bits), a hybrid approach combining predefined structural features with hash-based encoding (Gedeck et al., 2006); PubChem (881 bits), incorporating hierarchical structural features across seven categories (Kim et al., 2016); Atom Pair (2048 bits), representing atom pair fragments through distance-based hashing (Carhart et al., 1985); and Morgan (2048 bits), encoding circular atomic environments through hashed topology (Zhong and Guan, 2023).

#### Physicochemical properties

Physicochemical properties were primarily predicted using the Open Structure-activity/property Relationship Model (OPERA) (Mansouri et al., 2018). For molecules that could not be predicted with OPERA (Compounds fall outside OPERA’s applicability domain when their molecular weight, chemical structure, or physicochemical properties differ significantly from the training data used to develop the predictive models), VP and Water solubility (WS) were predicted using ChemBCPP (Dong et al., 2017), while LogP values were obtained from DataWarrior (Sander et al., 2015). Only 58% of all compounds could be predicted by OPERA (679/1174).

To ensure consistency across multiple predictive tools, all vapor pressure (VP) and water solubility (WS) values were converted to log mmHg and log mol/L, respectively, matching the ChemBCPP output format. This standardization was necessary because ChemBCPP was used to generate missing values for compounds that could not be parsed by the primary software. LogP values obtained from DataWarrior were already expressed as Calculated LogP (ClogP) and did not require conversion. A correlation assessment confirmed high concordance between the tools (Supplementary Figure S1).

Additional molecular descriptors, including molecular weight, topological polar surface area (TPSA), and structural features such as presence of conjugation, ring structure or ring counts, were calculated using RDKit cheminformatics toolkit.

#### Structural alert (Toxtree)

Structural alerts were generated using the skin sensitization decision tree in Toxtree desktop program v3.10, developed by VEGA (Patlewicz et al., 2008).

Toxtree employs a mechanistic approach to predict skin sensitization by identifying specific electrophilic moieties, such as SNAR, SN2, Michael acceptors, Schiff bases, and acyl transfer agents, which are associated with skin sensitization potential. This differs from read-across methods, which rely on chemical similarity-based rules. For the entire dataset (n = 1174), Toxtree achieved an overall accuracy of 65% (759/1174), with a false positive rate of 11% (134/1174) and a false negative rate of 24% (281/1174). Analysis of structural characteristics revealed that while the proportion of conjugated compounds and cyclic compounds remained almost consistent between correctly and incorrectly predicted compounds, multi-ring structures presented specific challenges. Of the 287 compounds containing two or more rings, 114 were mispredicted (40%). Notably, 75% of these mispredictions (86/114) were false negatives, indicating a tendency for Toxtree to systematically underpredict sensitization potential in multi-ring structures.

The best performing Michael acceptor achieved only 18% accuracy, and other alerts like Acyl transfer, Schiff base formation, and SN2 showed even lower accuracy. Notably, the SNAR alert had the worst performance, with only 2.4% accuracy. While Toxtree achieved an overall accuracy of 65% in this dataset, this was primarily driven by the accuracy of compounds without any Toxtree alerts (“no alert”). Therefore, while the lack of structural alerts likely indicates a lower probability of being a skin sensitizer, the presence of a structural alert is not necessarily a good predictor (Alves et al., 2016; 2018), leading to the necessity of more sophisticated approaches.

### Chemical similarity

The chemical similarity index was calculated for each of the fingerprints using RDKit’s Tanimoto similarity function, except for PubChem, which was done using the PubChem score matrix service: the PubChem chemical identifier (CID); a chemical identifier specific to PubChem was supplied to the score matrix, and similarity was then calculated using the 2D PubChem fingerprint and Tanimoto distance between the chemical fingerprints. The score matrix or similarity scores matrix reproduced from all the fingerprint approaches was then converted to a pairwise distance on Google Colaboratory in Python. A similarity cut-off of ≥70% and above was then used for further analysis.

### Modeling

#### Training

We employed an integrative, hierarchical training approach to assess the impact of feature stacking on model performance. Models were developed using three cumulative feature sets: fingerprints alone, fingerprints with structural alerts, and a fully integrated set adding physicochemical properties. This design enabled the quantification of the added predictive value offered by toxicologically relevant features compared to a structural baseline.

#### Training and test preparation

The dataset was split into training (80%) and test sets (20%) using a stratified random split function in Python. To match the units predicted by ChemBCPP (water solubility in log mol/L and VP in log), all physicochemical properties generated by OPERA were log-transformed and subsequently standardized using the scikit-learn StandardScaler (Dong et al., 2017).

### Models

#### K-nearest neighbor (KNN)

The use of the KNN model (Cover and Hart, 1967) a simple and reliable supervised machine learning model (Cosenza et al., 2021), in read-across is well documented (Chavan et al., 2015; Luechtefeld et al., 2016). The KNN model is a non-parametric classifier that predicts the class label of a new compound by identifying its k-nearest neighbors in the training set based on distance metrics. The prediction is determined by majority vote among these nearest neighbors. In this work, KNN was utilized to estimate the target outcome (skin sensitization status) based on the quantitative structural relationships encoded in molecular fingerprints, structural alerts, and physicochemical properties. We employed weighted KNN models with the Euclidean distance metric for classification. We manually varied the number of neighbors (k) from 3 to 7, training and testing the models on the dataset (n = 939 for training, n = 235 for testing) for each fingerprint approach across all domains.

#### Random forest

The RF model is a supervised ensemble model for classification and regression problems developed by Leo Breiman (Breiman, 2001). Its use for the development of *in silico* tools, particularly for skin sensitization hazard assessment, has been historically established (Luechtefeld et al., 2015). In brief, the RF model implementation (Pedregosa et al., 2011) version 1.3.1 was trained separately on the training set (n = 939), which had individual fingerprint methods combined with all physicochemical properties (LogP, WS, VP) and Toxtree alerts. We manually varied the number of trees (n_estimators) between 40 and 55 including the random state between 1 and 33 for each fingerprint approach. More information about these models can be found in (Marsland, 2009). All models were developed using the Python scikit-learn framework.

We selected model parameters based on prior studies (Golden et al., 2021; Koutroumpa et al., 2025; Scheufen Tieghi et al., 2024) and their widespread use in regulatory toxicology to ensure consistency and practical relevance. Given the comparative nature of this study, parameter were also chosen to allow each model to perform near optimally while maintaining a fair and controlled evaluation.

### Model evaluation

The models were evaluated on the test set using common classification metrics, including accuracy, precision, recall, F1-score, and balanced accuracy. Accuracy is the ratio of correct predictions to the total number. Precision measures the proportion of true positives among all predicted positives. Recall indicates the fraction of true positives identified by the model. The F1-score is a weighted average of precision and recall. Balanced accuracy (BA) represents the average of specificity and sensitivity.

Sensitivity (Sn), the true positive rate, and specificity (Sp), the true negative rate, are statistically based metrics for classification models. They measure the ability to distinguish between positive and negative classes, in this case, sensitizers and non-sensitizers (Ballabio et al., 2007; Todeschini, 1989). Sn and Sp are calculated using a confusion matrix, a table summarizing the observed and predicted classes comprised of the true positive (TP), true negative (TN), false negative (FN) and false positive (FP).

Accuracy is expressed as

$$Accuracy=\frac{TP+TN}{TP+TN+FP+FN}$$

Balanced accuracy is expressed as:

$$BA=\frac{\left(Sn+Sp\right)}{2}$$

Sensitivity (Sn) is expressed as:

$$Sn=\frac{TP}{TP+FN}$$

Specificity (Sp) is expressed as:

$$Sp=\frac{TN}{TN+FP}$$

F1 is expressed as:

$$F1=2X\;\frac{Precison\;X\;Recall\;}{Precision+Recall}$$

Precision is expressed as:

$$Precision=\frac{TP\;}{TP+FP}$$

Recall is expressed as:

$$Recall=\frac{TP\;}{TP+FN}$$

#### Feature importance

To identify the key features contributing to the best performing model predictions, we employed SHAP (Shapley Additive Explanations) analysis using its Python implementation (version 0.42.1). SHAP is a game-theory-based approach that assigns each feature an important value for a particular prediction by calculating the contribution of each feature to the difference between the actual prediction and the average prediction across the dataset. More information about SHAP is available here (Lundberg and Lee, 2017). SHAP summary plots were generated to investigate feature importance based on their mean absolute SHAP values, providing insight into the most influential variables across modeling approaches.

#### Comparison with other models

To assess the predictive performance of our approach against prominent skin sensitization models using the test set (n = 235). We performed a comparative analysis with three established tools: Toxtree, a rule-based classifier relying on structural alerts; and two Quantitative Structure-Activity Relationship (QSAR) models CAESAR (v2.1.7), IRFMN (v1.0.1) (Baldoni et al., 2013) implemented within the VEGA suite (v1.2.5) and SbD4Skin by EosCloud (Koutroumpa et al., 2025). The VEGA platform provides a comprehensive collection of *in silico* models for diverse toxicity endpoints, while SbD4Skin employs a Multiview molecular representation approach for predicting skin sensitization, irritation, and acute dermal toxicity. Their skin sensitization modules were chosen for analysis. Both platforms incorporate quantitative reliability metrics, including Applicability Domain Index (ADI), to evaluate prediction confidence. CAESAR and IRFMN models were accessed through the VEGA suite, and all predictions, including those from SbD4Skin, were generated in batch mode.

#### Applicability domain assessment

The applicability domain (AD) of the model was assessed using a similarity-based approach based on Tanimoto similarity between molecular fingerprints adapted from (Koutroumpa et al., 2025). This methodology evaluates whether test compounds fall within the chemical space represented by the training set, providing a measure of prediction reliability. Avalon fingerprints (2048 bits) were computed for all compounds using RDKit. A similarity threshold (S_T) was calculated from the training set according to

$$S\_T=\bar{\gamma }+Z\sigma$$

where $\bar{\gamma }$ represents the average pairwise Tanimoto similarity between all training compounds, σ is the standard deviation of these similarities, and Z is a predefined parameter controlling the stringency of the AD boundary. A Z value of 0.5 was used, representing a liberal threshold that balances coverage with reliability. For each test compound, the Tanimoto similarity to all training compounds was computed. The average similarity to the k most similar training compounds (k = 5) was then compared to the threshold S_T. A test compound was considered within the AD if its average similarity to its k nearest neighbors exceeded the threshold, indicating sufficient structural similarity to the training set for reliable predictions. Compounds falling outside the AD were flagged as having potentially less reliable predictions due to their structural dissimilarity to the training data. The AD coverage was visualized using t-distributed stochastic neighbor embedding (t-SNE) to project the high-dimensional fingerprint space into two dimensions, allowing assessment of the chemical space overlap between training and test sets.

## Results

### Dataset

**FIGURE 1:**
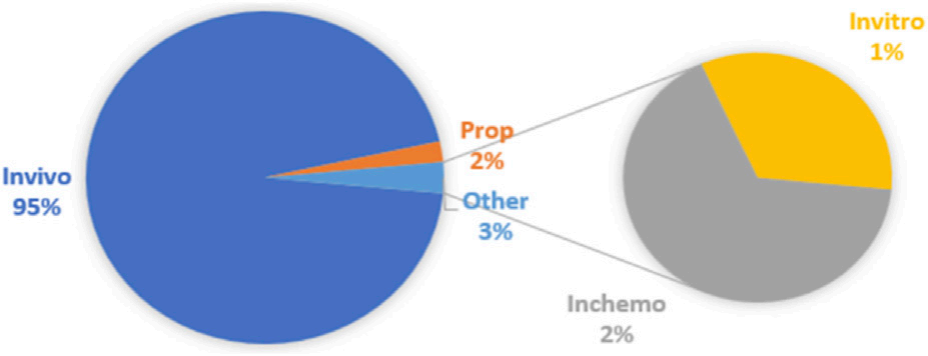
Data distribution: Pie chart shows that 95% of the dataset were from *in vivo* source, 2% were provisional datasets from the USA EPA high production volume chemical database which served as provisional negative while 3% were from other sources, namely, *in chemo* source and *In vitro* sources from animal studies, human patch test studies.

The dataset used in this study is summarized in (Supplementary Figure S2). Data were compiled from multiple sources, as described in Methods section, primarily from *in vivo* studies (LLNA, Buehler’s test, and a small portion from human patch tests; see Figure 1). These data were balanced to reduce bias (see Data section in methods), resulting in 1174 chemicals. Of these, 656 (55.9%) were classified as sensitizers and 518 (44.1%) as non-sensitizers. While the dataset showed good concordance with harmonized CLP skin sensitization classifications, only 26 high-production-volume chemicals lacking manufacturer sensitization data remained as provisional negatives after metal and metal salts were excluded. Although they lacked manufacturer-provided sensitization data, 25 of these 26 provisional negatives had CLP classifications for non-sensitizers, supporting their provisional status.

## Performance

### Chemical similarity

Other approaches to predicting skin sensitization (e.g., the OECD QSAR Toolbox) focus on using structurally similar compounds. However, defining “similar” is not straight-forward, and is in fact highly dependent on the choice of fingerprint method as well as the chosen Tanimoto cut-off. Using a similarity cut-off of ≥70%, PubChem demonstrated the highest number of pairwise relationships compared to other substructure-based fingerprints, such as MACCS and Avalon (Table 1). This is primarily due to their longer bit length (881 bits), which allows them to encode a broader and more diverse range of structural features. As a result, PubChem is more likely to share bits between different compounds, effectively increasing the frequency of detected similarities. In contrast, MACCS and Avalon fingerprints identified fewer similar chemicals, while Morgan and Atom Pair FP showed the smallest number of pairwise relationships.

**TABLE 1:** Molecular fingerprint analysis (Tanimoto similarity and fingerprint design).

Fingerprint	Number of similar pairs (≥70%)	Mean similarity (%)	FP count	Design	Class
Morgan	23,040	10	2048	Sparse	Circular
Pubchem	303,804	27	881	Dense	Substructure
Avalon	39,192	13	512	Dense	Substructure
Maccs	127,848	21	167	Dense	Substructure
Atom pair	17,448	10	2048	Sparse	Atom pair

This table summarizes the number of similar compound pairs with Tanimoto similarity greater than or equal to 70%, the mean similarity between compounds produced by each fingerprint, the fingerprint bit count, the molecular fingerprint design, and molecular fingerprint class, which provide insight into how molecular constituents are annotated or encoded by each fingerprint method. Design refers to bit density: dense fingerprints (MACCS, PubChem, Avalon) have higher proportions of non-zero bits due to dictionary-based pattern matching, while sparse fingerprints (Morgan, Atom Pair) have mostly zero bits due to hash-based encoding. Class indicates the encoding methodology: circular (atom neighborhood-based), substructure (predefined pattern-based), or atom pair (distance-based).

### KNN model

**FIGURE 2:**
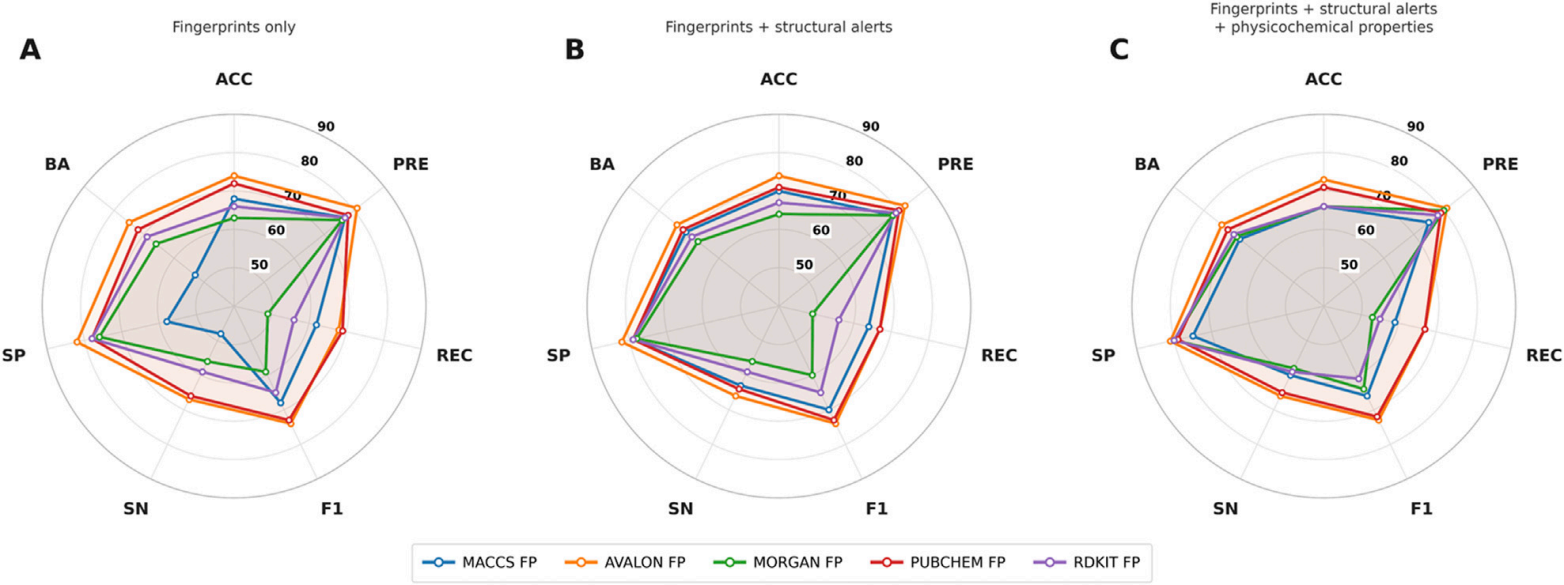
KNN model performance across different feature combinations. Radar plots display seven performance metrics: specificity (SP), sensitivity (SN), F1-score (F1), recall (REC), precision (PRE), balanced accuracy (BA), and accuracy (ACC). Different colored lines represent different fingerprint types. **(A)** KNN performance using molecular fingerprints only, showing variable performance across different fingerprint methods. **(B)** Enhanced and more consistent performance when structural alerts are integrated with molecular fingerprints, with most fingerprint types showing improved metrics. **(C)** Mixed results when physicochemical properties are added to the fingerprint-structural alert combination, with some fingerprint methods showing performance degradation, particularly in sensitivity and specificity.

The optimal number of neighbors for this dataset was k = 4, since increasing k led to underperformance. For detailed performance with other k values, please refer to (Supplementary Tables S1-S15). KNN models with fingerprints alone achieved accuracy scores of 63%–73% and balanced accuracy rates of 69%–74% depending on choice of fingerprint. Integrating structural alerts with fingerprints slightly reduced accuracy (64%–71%) while maintaining balanced accuracy (69%–74%). Adding physicochemical properties had a minimal impact on performance (Figure 2).

Avalon fingerprint consistently outperformed other approaches, even though it did not have a high number of structurally similar pairwise relationships. However, there was no straightforward connection between the number of similar compounds and performance as PubChem, with a higher number of relationships, outperformed MACCS, which in turn outperformed Morgan and Atom Pair FP.

Integrating additional features like structural alerts and physicochemical properties into KNN led to a decline in performance across all approaches and domains. This decline in performance suggests the limitations of KNN in handling complex, high-dimensional data. Further analysis using 10-fold cross-validation on the complete feature integration domain which included fingerprints, structural alerts, and physicochemical properties yielded accuracy scores ranging from 59% to 68%, precision scores of 62%–69%, recall scores of 61%–69%, and F1-scores between 58% and 68%. A detailed summary of these results is provided in (Supplementary Table S16).

### Random Forest model

**FIGURE 3:**
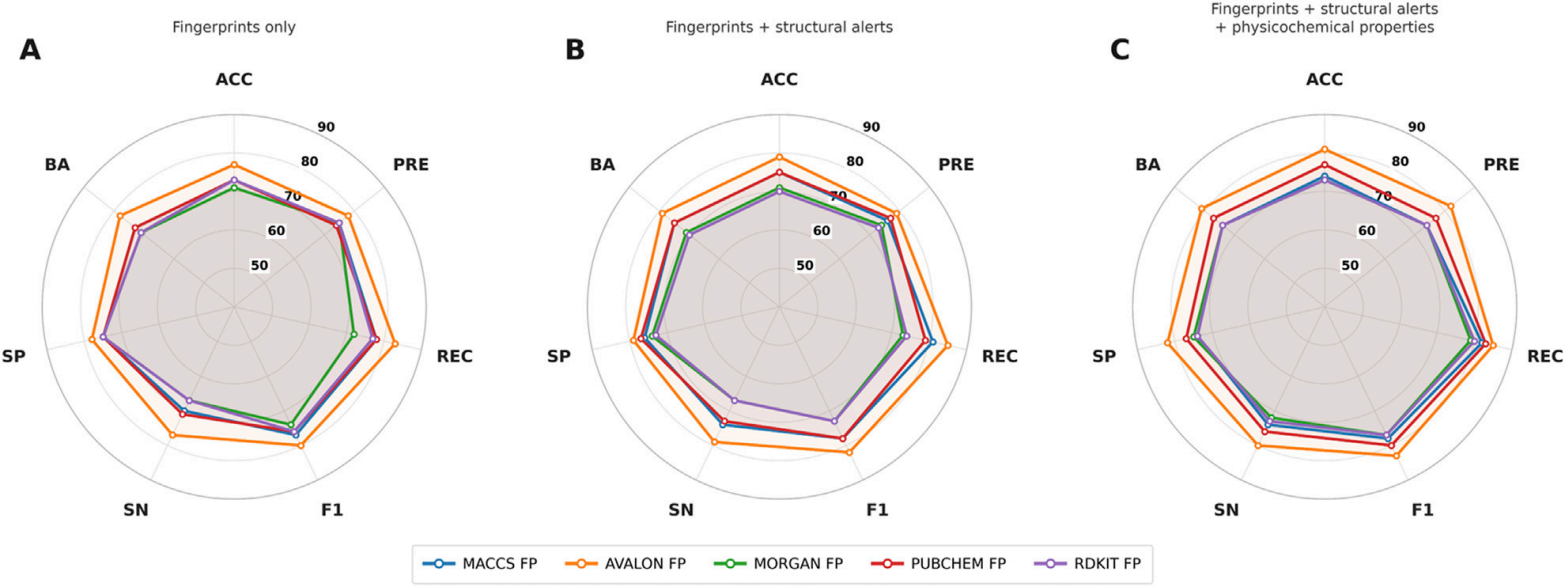
RF model performance across different feature combinations. Radar plots display seven performance metrics: specificity (SP), sensitivity (SN), F1-score (F1), recall (REC), precision (PRE), balanced accuracy (BA), and accuracy (ACC). Different colored lines represent different fingerprint types. **(A)** RF performance using molecular fingerprints only with Avalon achieving notably higher performance across most metrics compared to other fingerprint methods. **(B)** Performance when structural alerts are integrated with molecular fingerprints, showing more balanced performance across fingerprint types with reduced variation between methods. **(C)** Results when physicochemical properties are added to the fingerprint-structural alert combination, demonstrating maintained high performance for the top-performing fingerprint while other methods show mixed results.

Training and testing the RF model with just the fingerprint approach yielded accuracy scores ranging from 71% to 77% and balanced accuracy from 71% to 78%. Integrating fingerprints with a structural alert resulted in accuracy scores of 70%–79% and balanced accuracy of 70%–79%. When all features (fingerprints, structural alert, and physicochemical properties) were integrated, accuracy scores ranged from 71% to 81% and balanced accuracy from 74% to 81% (Figure 3). As observed with KNN, the Avalon fingerprint consistently outperformed all others across all evaluation metrics in the third domain. Further analysis with 10-fold cross-validation on the complete feature integration (fingerprints, alert, and physicochemical properties) revealed accuracy scores between 70% and 74%, precision scores of 70%–74%, recall scores of 69%–73%, and F1-scores of 69%–73%. These results are summarized in (Supplementary Table S17).

Compared to KNN, RF models consistently performed better (Table 2). This advantage was evident across all approaches and domains. Notably, RF models trained solely on fingerprints surpassed KNN’s performance. This improvement was further amplified when structural alerts were integrated, and even more so when all features (fingerprints, structural alerts, and physicochemical properties) were combined. The fact that integrating structural alerts and physicochemical properties with fingerprints led to improved performance in the RF models strongly suggests that these features hold valuable information for predicting skin sensitization (Supplementary Tables S18-S20).

**TABLE 2:** Performance comparison of best-performing models on test set versus 10-fold cross-validation.

Model	ACC(%)	PRE(%)	Recall (%)	F1 (%)
AVALON_RF	81	82	85	83
AVALON _KNN	73	81	67	73
AVALON_RF_CV10	74	74	73	73
AVALON_KNN_CV10	67	68	68	68

Performance comparison of Random Forest (RF) and K-Nearest Neighbors (KNN) models using Avalon fingerprints with complete feature integration. Test set performance (n = 235) and 10-fold cross-validation (CV10) results shown. ACC, accuracy; PRE, precision; RECALL, sensitivity; F1 = F1-score. All values expressed as percentages.

Previous studies have demonstrated that fingerprint design can influence both molecular similarity and performance (Kuwahara and Gao, 2021; Mellor et al., 2019; Safizadeh et al., 2021). However, in this case, we could not definitively conclude which fingerprint was the best. Notably, fingerprints with shorter bit lengths (Avalon, PubChem, and MACCS) generally outperformed those with longer bit lengths. This could be attributed to these fingerprints being substructural fingerprints and their ability to focus on capturing essential molecular features without overcomplicating the representation. For instance, Avalon could have outperformed due to its design, which combines predefined structural features with hash-based encoding. This suggests that combining the interpretability of dictionary-based features with the flexibility of hash-based encoding may be optimal for skin sensitization prediction. Pure dictionary-based fingerprints (PubChem, MACCS) showed intermediate performance, while pure hash-based approaches (Morgan, Atom Pair) performed poorly.

### Misprediction

Conjugated systems characterized by connected *p*-orbitals, alternating single and multiple bonds, or lone pairs facilitate electron delocalization, thereby influencing structural stability and chemical reactivity; these features are directly implicated in skin sensitization (Delaine et al., 2013; 2014). Mechanistic studies indicate that conjugation is often a prerequisite for certain moieties to elicit a sensitization response. For example, reactive structures such as aldehydes or epoxides often require conjugation (e.g., in alpha and beta unsaturated systems) to act as sensitizers, largely because this electronic configuration facilitates the covalent binding necessary for haptenization, either directly or following bioactivation or autooxidation. Consequently, conjugated unsaturated moieties are frequently associated with strong sensitization potential, whereas their unconjugated analogues often exhibit weak or non-sensitizing effects (Delaine et al., 2013; Karlberg et al., 2008). Similarly, the cyclicity of a compound plays a critical role in determining sensitization potency. The distinction between aromatic and non-aromatic cyclic structures significantly alters the metabolic fate and reactivity of the parent compound (Dascalu et al., 2023). Furthermore, these structural features fundamentally govern the physicochemical properties of the molecule, such as molecular size, hydrophobicity (LogP), and degree of saturation, which in turn determine bioavailability and penetration (Agatonovic-Kustrin et al., 2020). Likewise, structural alerts represent predefined electrophilic functional groups or moieties that serve as mechanistic indicators of toxicity. In the context of skin sensitization, these alerts correspond directly to the Molecular Initiating Event (MIE) of the Adverse Outcome Pathway (Ta et al., 2021). Crucially, however, sensitization potential is not determined solely by the presence or absence of an alert; their abundance within the overall molecular structure has been shown to exert differential influences on both biological potency and model predictivity (Golden et al., 2023). Given their critical role in skin sensitization, it was important to determine whether mispredictions occur randomly or are systematically associated with specific chemical features.

We analysed misprediction patterns across specific compound characteristics particularly conjugated systems, cyclic structures, and structural alerts comparing them to both overall dataset and test set distributions. To assess the fundamental stability of the machine learning algorithms independent of feature representation, misprediction rates were averaged across all five fingerprinting approaches. This aggregation ensures that the reported patterns reflect the intrinsic biases of the RF and KNN algorithms rather than the artifacts of a single fingerprint method.

The overall dataset contained 46% (547/1174) conjugated compounds, 63% (744/1174) cyclic compounds (38% single-ring, 24% multi-ring), and 43% (507/1174) compounds with structural alerts. The test set exhibited nearly identical distributions: 45% (106/235) conjugated, 64% (151/235) cyclic, and 39% (93/235) with alerts. This concordance confirms the representative sampling approach and eliminates selection bias as a confounding factor.

Conjugated compounds were underrepresented in mispredictions (35% for both RF and KNN) relative to their prevalence in the dataset (46%). This suggests that despite abundant training examples, the models performed better on conjugated systems, likely due to their distinct electronic signatures. KNN false positives were predominantly non-conjugated (62%) rather than conjugated (38%). Similarly, RF false negatives were heavily skewed toward non-conjugated compounds (73%) compared to conjugated ones (27%), indicating a specific difficulty in predicting toxicity for non-conjugated structures. See (Supplementary Figure S3) for prediction distributions across models.

Cyclic compounds were also underrepresented in the misprediction, comprising 63% of the dataset but only 54% of mispredictions for both models. RF showed a pronounced bias in false negatives; 58% of missed sensitizers were non-cyclic, whereas multi-ring compounds accounted for only 8% of false negatives. KNN mispredictions were more distributed, though false negatives still skewed toward non-cyclic compounds (45%).

Compounds with multiple structural alerts were rare (6% of dataset, 5.1% of test set). In the analysis of mispredictions, the majority involved compounds without any alerts (55% RF, 53% KNN).

While compounds with single alerts showed variable misprediction patterns, compounds with two or more alerts revealed a critical systematic failure. These complex structures were predominantly classified as False Negatives by RF and exclusively as False Negatives by KNN, demonstrating a systematic underprediction of toxicity for structurally complex compounds, likely driven by insufficient training exposure to these rare, multi-reactive motifs (Supplementary Figures S4, S5).

### Activity cliffs

Activity cliffs (ACs) have historically represented a significant challenge for (QSAR) models. These phenomena are characterized by a discontinuity in the Structure-Activity Relationship (SAR) landscape, where structurally similar compounds often differing by minor modifications such as the addition of a hydrogen bond donor or acceptor near a binding site exhibit drastic differences in biological potency (Hu et al., 2013; Stumpfe et al., 2019).

While some approaches have proposed pruning ACs from datasets to smooth the biological landscape, this practice is problematic; removing cliffs may compromise model applicability and predictive power by discarding critical SAR information required for fine-grained discrimination (Dablander et al., 2023). Instead, efforts have focused on characterizing these landscapes using metrics such as the Modelability Index (MODI), which quantifies the smoothness of the binary classifier boundary and serves as a predictor of QSAR modeling success (Golbraikh et al., 2014). Similarly, it has been demonstrated that the prevalence of cliff-forming compounds in a dataset is inversely correlated with model performance (Sheridan et al., 2020).

To address these challenges, diverse methodological frameworks have been explored using non-skin sensitization data set. Traditional machine learning algorithms including K-Nearest Neighbors (KNN), Random Forest (RF), and Support Vector Machines (SVM) have been extensively applied, particularly when integrated with physicochemical properties or Extended Connectivity Fingerprints (ECFPs) including graph-based representations (Dablander et al., 2023). More recent advancements have introduced non-linear deep learning architectures, such as Multi-Layer Perceptron (MLP), Graph Neural Networks (e.g., Message Passing Neural Networks, Graph Convolutional Networks), and SMILES-based transformers like ChemBERTa (van Tilborg et al., 2022). However, comparative benchmarks indicate that increased algorithmic complexity does not guarantee superior performance in this domain. Studies have shown that traditional algorithms (e.g., SVM and RF) often outperform or match advanced deep learning methods in AC prediction, particularly when robust descriptors like ECFPs are employed. Notably, integrating traditional models with molecular graph representations has also been documented to enhance the resolution of these activity discontinuities (Dablander et al., 2023).

For skin sensitization, these have been evaluated using PubChem fingerprints and KNN, a combination that has demonstrated limited predictive performance (Golden et al., 2023).

Here, we leverage chemical similarity maps to visualize how different fingerprint methods define similarity within the chemical space of our dataset. Analyzing these maps allows us to identify patterns that explain why some methods outperform others from a chemical similarity perspective. Ideally, when defining chemical similarity, clusters of structurally related compounds should share the same sensitization status; however, this is often not the case, as chemical clusters frequently exhibit discordant biological activity. Critically, the ability to detect these clusters depends on the density of the network. Consequently, molecular fingerprint selection played a decisive role in how chemical space was analyzed. While Morgan and Atom Pair fingerprints were initially considered, they yielded a limited number of pairwise relationships between compounds, resulting in sparse networks that were insufficient for robust cluster analysis, hindering the exploration of chemical space for insights. Consequently, Avalon, PubChem, and MACCS fingerprints were chosen for their ability to generate networks of pairwise relationships. This representation of chemical space facilitated the investigation of potential performance-driving patterns within RF and KNN models.

Analysis of RF models revealed that chemical similarity maps exhibited distinct topologies depending on the fingerprint used. PubChem FP and MACCS FP produced large, comprehensive global maps, whereas Avalon FP generated smaller, more discrete clusters. These topological differences influenced how mispredictions were distributed and how readily misprediction patterns could be identified.

**FIGURE 4:**
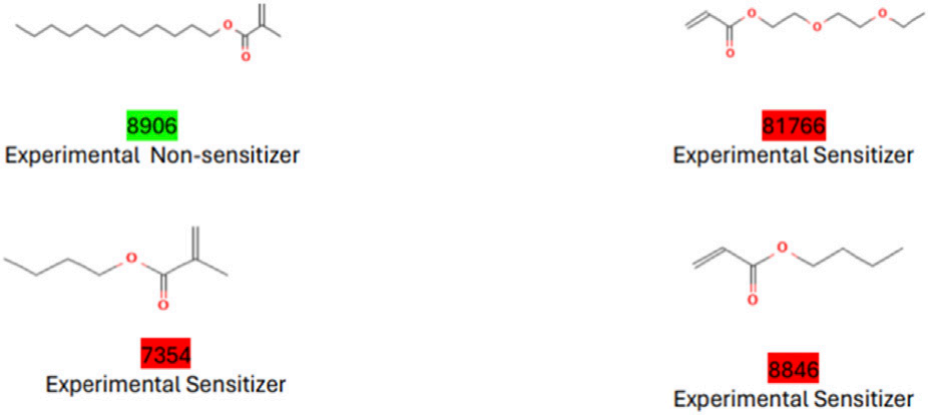
Resolution of activity cliffs among structurally similar acrylates (Tanimoto similarity ≥ 70%). The cluster compares the non-sensitizer 8904 (Dodecyl methacrylate) against sensitizer analogs 81766 (2-(2-Ethoxyethoxy)ethyl acrylate), 7354 (Butyl methacrylate), and 8846 (Butyl acrylate). Color Key: Green = Non-sensitizer, Red = Sensitizer. While Toxtree and KNN models incorrectly classified the non-sensitizer (8904) as active (False Positive), the Avalon-RF model accurately predicted the sensitization status for all compounds in the cluster.

**FIGURE 5:**
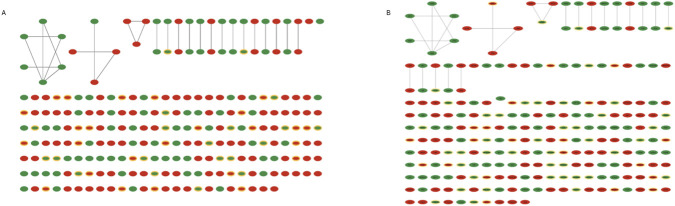
Avalon fingerprint similarity networks comparing RF and KNN performance. Chemical similarity network analysis comparing Avalon-RF and Avalon-KNN performance on structurally similar compounds (Tanimoto similarity ≥70%). Node colours indicate ground truth classification: green = non-sensitizer, red = sensitizer. Yellow ellipses highlight mispredictions (false positives or false negatives). Isolated nodes represent compounds without structural neighbours above the similarity threshold. **(A)** Avalon-RF correctly classifies all compounds across three distinct cluster types: concordant sensitizers (Cluster 1), mixed sensitizer/non-sensitizer pairs representing activity cliffs (Cluster 2), and concordant sensitizers (Cluster 3). **(B)** Avalon-KNN mispredicts the non-sensitizer in Cluster 2 (false positive) and one sensitizer within Cluster 3 (false negative), demonstrating RF’s superior ability to handle activity cliffs among structurally similar compounds.

The Avalon-RF model demonstrated strong performance within its discrete cluster structure. In Cluster 2, three structurally similar sensitizers and one non-sensitizer all shared a Michael acceptor alert; the model correctly predicted all four compounds (Figure 4). Similarly, Cluster 3 contained three experimentally confirmed sensitizers lacking any Toxtree alerts, all of which were correctly predicted by Avalon-RF (Figure 5A).

**FIGURE 6:**
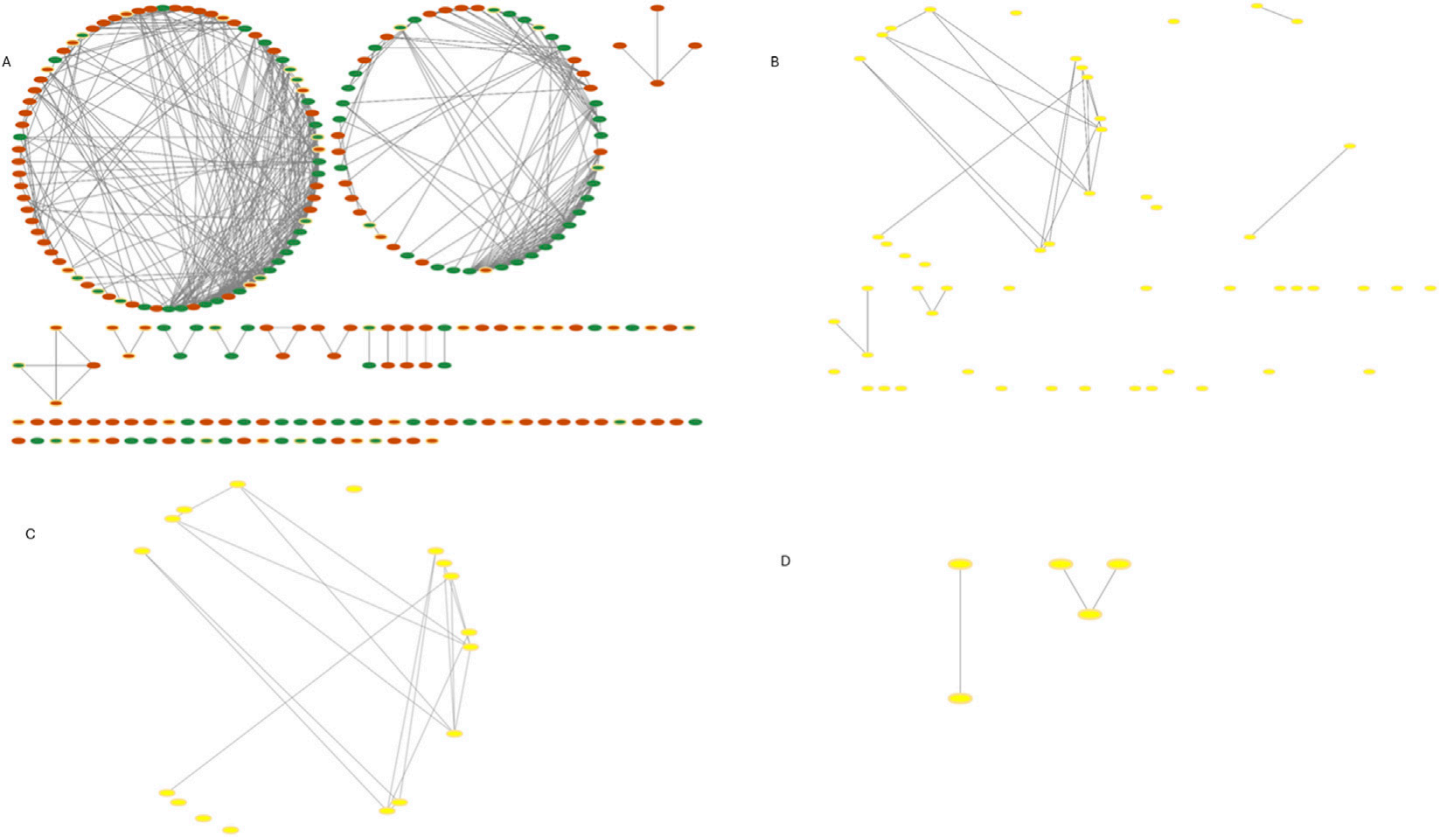
Chemical similarity network analysis of PubChem RF predictions on the test set. PubChem RF chemical similarity network analysis (Tanimoto similarity ≥70%). Node colors: green = non-sensitizer, red = sensitizer; yellow ellipses indicate mispredictions (false positives or false negatives). **(A)** Global network displaying all structurally similar compounds with prediction outcomes. **(B)** Filtered subnetwork showing only mispredicted compounds and their structural neighbors. **(C)** mispredicted compounds enriched in conjugated benzenoid structures, demonstrating topological clustering of prediction errors within this chemical class. **(D)** mispredicted compounds sharing Michael acceptor structural alerts, indicating that compounds with this reactive motif cluster together in regions of high prediction uncertainty.

The larger global maps from PubChem-RF and MACCS-RF presented greater prediction challenges, particularly at activity cliffs, instances where structurally similar compounds have discordant experimental outcomes. In the PubChem-RF map, mispredictions frequently occurred near such activity cliffs. Cluster 1 exemplified this pattern, with 67% of mispredictions involving conjugated compounds. Both conjugated systems and cyclic structures showed topological clustering, suggesting these features may be associated with prediction difficulties when compounds possessing them are positioned near activity cliffs (Figures 6A–D).

**FIGURE 7:**
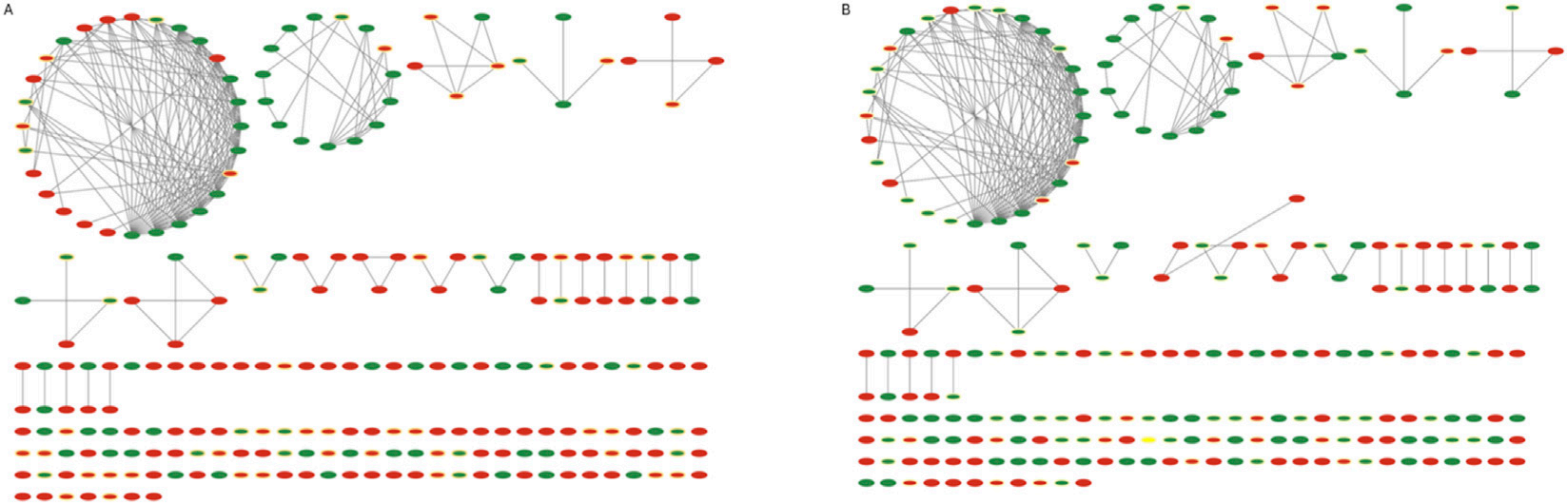
MACCS FP similarity networks showing clustering patterns and mispredictions. Comparison of MACCS-RF and MACCS-KNN predictions across chemical similarity clusters (Tanimoto similarity ≥70%). Node colors: green = non-sensitizer, red = sensitizer; yellow ellipses indicate mispredictions. **(A)** MACCS-RF: 12 clusters identified; 9 clusters (75%) contain at least one misprediction, with 6 clusters (50%) containing ≥2 mispredictions. Cluster 7 represents the only cluster where the model correctly distinguished structurally similar compounds with discordant activities. **(B)** MACCS-KNN: 12 clusters identified; 11 clusters (92%) contain at least one misprediction, with 7 clusters (60%) containing ≥2 mispredictions, demonstrating poor performance compared to RF in resolving activity cliffs.

MACCS-RF produced 12 distinct clusters, 11 of which contained a misprediction. Mispredictions typically occurred between structurally similar compounds with different sensitization outcomes. Cluster 7 was a notable exception where the model correctly distinguished structurally similar compounds with discordant activity, though this success was overshadowed by high misprediction rates in other clusters with similar structural characteristics (Figure 7A). For both Avalon-RF and MACCS-RF, mispredictions were distributed across many small clusters rather than concentrated in a few large ones, limiting the ability to identify systematic misprediction patterns.

**FIGURE 8:**
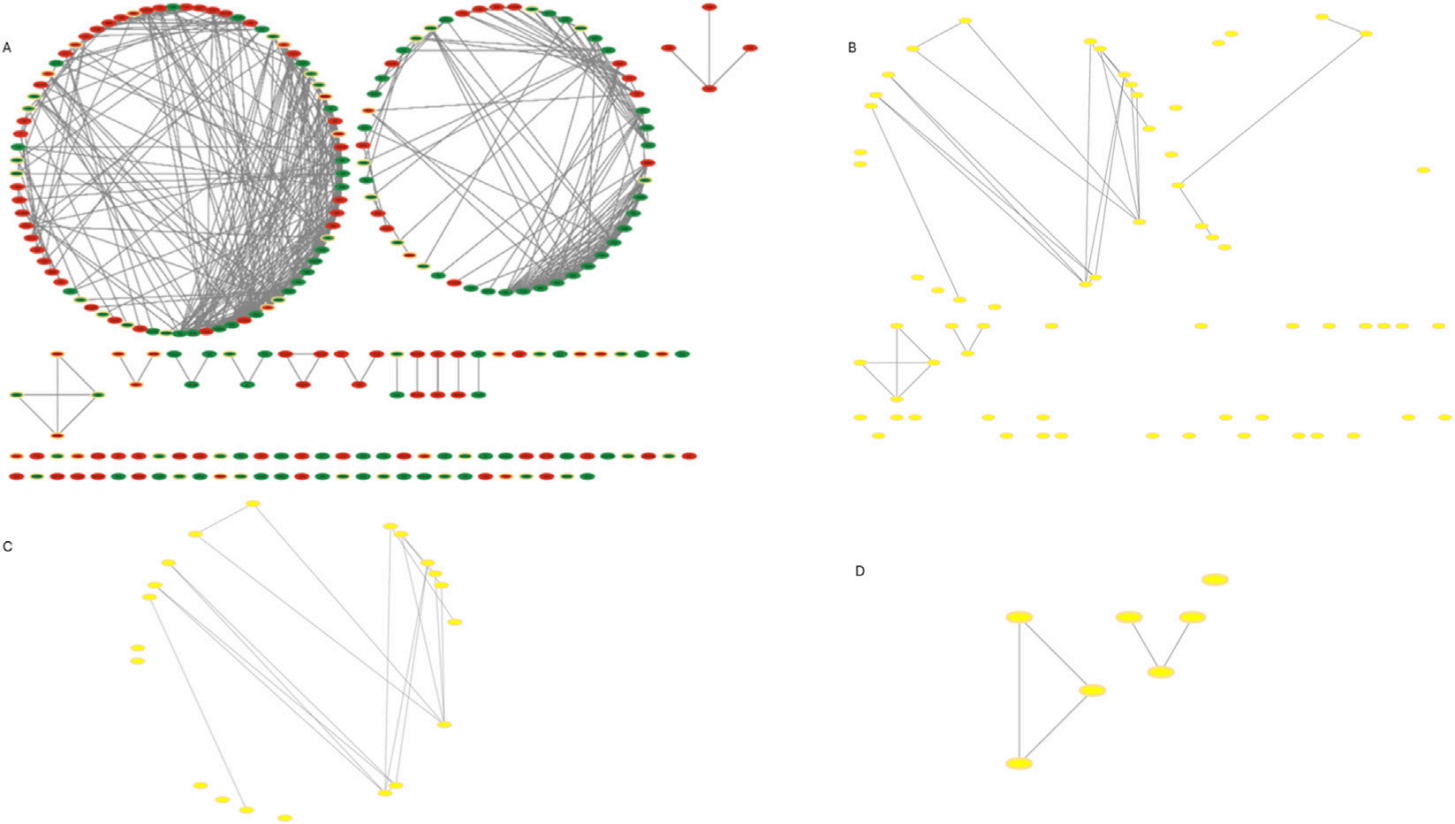
Chemical similarity network analysis of PubChem KNN model predictions. PubChem-KNN chemical similarity network analysis (Tanimoto similarity ≥70%). Node colors: green = non-sensitizer, red = sensitizer, yellow = misprediction. **(A)** Global similarity network displaying all structurally related test compounds and prediction outcomes. **(B)** Filtered subnetwork containing only mispredicted compounds and their immediate structural neighbors, revealing spatial clustering of misprediction. **(C)** Predominantly conjugated aromatic compounds, where mispredictions cluster among structurally similar benzenoid derivatives. **(D)** Compounds sharing Michael acceptor structural alerts; despite common electrophilic functionality, these non-sensitizers were incorrectly predicted as sensitizers, demonstrating that shared reactive motifs do not ensure prediction consistency.

KNN models were generally outperformed by their RF counterparts across all fingerprints. This performance gap was evident with Avalon-KNN, which failed to achieve accurate predictions for compounds in Clusters 2 and 3 where Avalon-RF had succeeded (Figure 5B). PubChem-KNN produced unique misprediction clusters, including one where compounds sharing a Michael acceptor alert were incorrectly predicted as sensitizers despite being experimentally negative; these compounds were located near other clusters with high frequencies of similar structural alerts (Figures 8A–D). MACCS-KNN showed the most pronounced limitations: 11 of 12 identified clusters contained at least one misprediction, and nearly 60% contained two or more mispredicted compounds (Figure 7B).

### SHAP analysis

**FIGURE 9:**
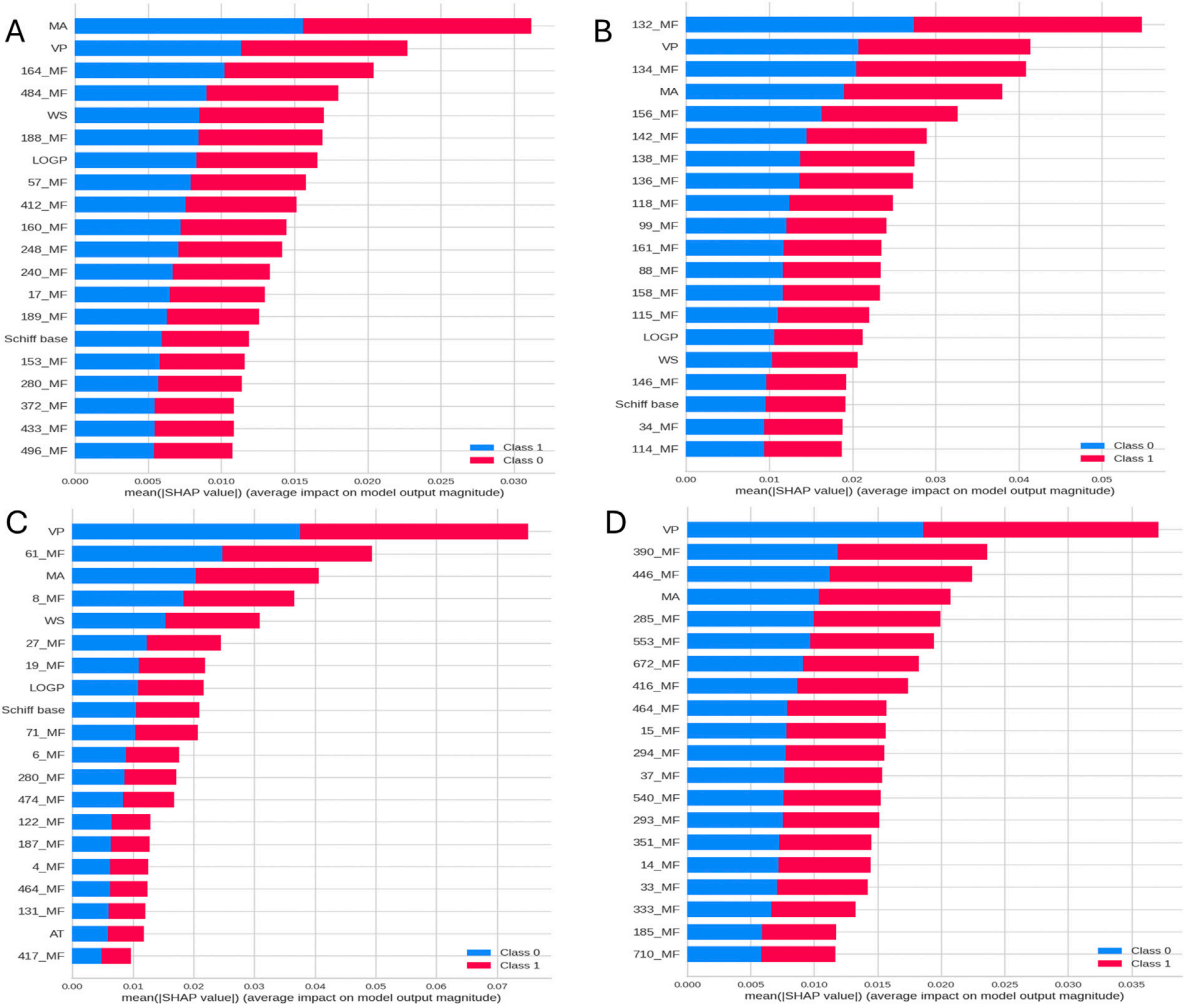
SHAP feature importance analysis across different fingerprint methods. Horizontal bar plots show the mean SHAP values (average impact on model output) for the top molecular and non-molecular features. Blue bars represent contributions toward class 0 (non-sensitizers), red bars represent contributions toward class 1 (sensitizers). Features are ranked by total importance magnitude. **(A)** Atom Pair fingerprint features. **(B)** MACCS FP features. **(C)** Morgan fingerprint features. **(D)** PubChem fingerprint features.

Following the demonstration that RF models outperform KNN, the Python package for SHAP analysis was employed to identify key features driving predictions within the RF model. Notably, the number of important features identified by SHAP differed between approaches, likely due to variations in the molecular fingerprints used for each approach. SHAP analysis identified 20 key features across all modeling approaches (Figure 9). The features were grouped into structural (molecular) and non-structural (i.e., physicochemical properties and the presence or absence of a Toxtree structural alert) categories to disentangle their relative influence on the model’s prediction. Analysis of SHAP-derived molecular features and non-molecular features revealed key substructure and non-substructure influencing the RF model’s prediction of skin sensitization across most approaches. Due to current limitations in mapping Avalon fingerprint bits to interpretable substructures within RDKit, Avalon FP was excluded from SHAP-based substructure visualization (Supplementary Figures S6).

#### Approach for molecular feature analysis

To investigate which chemical features drive model predictions, we analysed the functional groups represented among top-ranked SHAP features across all fingerprinting approaches. For each fingerprint method, important bits were mapped to their corresponding chemical substructures using RDKit visualization modules, and the frequency of specific functional groups among these high-importance features was quantified. This analysis identified functional groups with well-established mechanistic links to skin sensitization, including aldehydes and epoxides, which act as electrophilic reactive species capable of forming covalent bonds with skin proteins. Beyond these known reactive groups, nitrogen-containing functionalities were the most frequently represented among important features (33.9%), followed by oxygen-containing groups (19.1%), sulfur-containing moieties (13.1%), and halogenated compounds (13.1%). These findings align with previous studies demonstrating the mechanistic relevance and predictive importance of these functional groups for skin sensitization (Koutroumpa et al., 2025; Lee et al., 2011; Scheufen Tieghi et al., 2024; OECD, 2012) (Table 3).

**TABLE 3:** Summary of the molecular fingerprint features as analyzed by SHAP.

Fingerprints	Number of features	Halogens	Sulfur	Aliphatic	Oxygen	Nitrogen	Others
MACCS	15	1	2	9	2	4	0
Morgan	14	3	3	7	2	5	3
Pubchem	18	2	2	11	4	6	1
Atom pair	15	2	1	8	4	6	0

Frequency of chemical moiety types among top SHAP-ranked features across all fingerprinting approaches. Aliphatic hydrocarbons appeared in 56.1% of features on average, followed by nitrogen-containing (33.9%), oxygen-containing (19.1%), halogenated (13.1%), and sulfur-containing (13.1%) groups, while aromatic and heterocyclic (annotated as others) substructures were the least common.

Aromatic and heterocyclic structural features showed low representation among top-ranked SHAP features, comprising only 6.8% across all fingerprint methods. However, this varied substantially by fingerprint type: Morgan FP identified aromatic/heterocyclic features in 21% of its important bits, compared to 6% for PubChem FP and 0% for both MACCS FP and Atom Pair FP. This variation likely reflects differences in structural encoding rather than true feature importance. Morgan fingerprints use circular atomic environments that naturally capture ring topologies and heteroatom patterns, whereas MACCS keys rely on predefined substructures that may not include the specific aromatic or heterocyclic patterns relevant to this dataset. Atom Pair fingerprints encode distance-based atom relationships rather than ring connectivity, making them less suited for representing aromatic systems (Supplementary Tables S21-S24). The interpretation that low SHAP importance reflects encoding limitations rather than lack of predictive relevance is supported by the high prevalence of aromatic rings in the dataset (nearly 50% of compounds; Supplementary Figure S7). These findings show a methodological consideration: fingerprint selection can systematically obscure the contribution of certain structural classes to model predictions, even when those features are mechanistically relevant.

#### Non-fingerprint features

SHAP analysis identified six out of eight non-molecular features as informative. These features comprised two categories: structural alert features (Michael Acceptor, Acyl Transfer, SN2, SNAR, and Schiff Base) and physicochemical properties (vapor pressure, water solubility, and lipophilicity). SHAP analysis revealed VP as the most contributing physicochemical property across all approaches for the RF model’s prediction of skin sensitization. This consistency might be attributed to VP’s higher variance compared to WS and LogP in the dataset. A wider distribution of values (variance) around the mean could potentially give VP an advantage in influencing model prediction (Bouthillier et al., 2021) (Supplementary Figure S8).

SHAP analysis also revealed WS and lipophilicity (LogP) as important features for most fingerprint approaches, except PubChem FP. This exception might be due to outliers or lower data variability than VP. Lower variability can statistically influence feature contributions in machine learning models. In general, WS and LogP are known to impact skin sensitization through their effect on skin permeability and bioavailability (Roberts et al., 2008; Roberts and Williams, 1982). These features have also been found to contribute to prediction in a similar SHAP analysis for skin sensitization using Mordred descriptors with ghost filtering (Koutroumpa et al., 2025) (Table 4).

**TABLE 4:** Summary of the non-molecular fingerprint features and their contribution to model.

Fingerprint	VP	WS	LOGP	MA	SB	AT	SN2	SNAR
MACCS	●	●	●	●	●	X	X	X
Avalon	●	●	●	●	X	X	X	X
Morgan	●	●	●	●	●	●	X	X
Pubchem	●	X	X	●	X	X	X	X
Atom pair	●	●	●	●	●	X	X	X

The SHAP summary plot reveals key features influencing skin sensitization prediction across most fingerprint approaches (Where ● shows present and X shows absent). Vapor pressure (VP) stands out as the most prominent non-molecular contributor, followed by WS and LogP (except for PubChem). Among structural alerts, Michael Acceptor (MA) has the strongest impact, with Schiff Base (SB) being influential for most approaches (excluding Avalon and PubChem). Finally, Acyl transfer (AT) alert is only utilized by MorganFP and Atom PairFP.

SHAP analysis revealed variable importance of structural alerts across fingerprint methods (Table 4). Michael acceptor alerts were consistently identified as important features across all approaches, supporting their established role in skin sensitization. However, other mechanistic alerts showed fingerprint-dependent importance: Schiff base alerts contributed to Avalon, PubChem, and Atom Pair models but not to MACCS or Morgan models, while acyl transfer alerts appeared important only for Morgan and Atom Pair fingerprints. This variation suggests that alert importance is influenced by how different fingerprints encode surrounding molecular context. Thus, while structural alerts provide mechanistically relevant information value, their contribution to model predictions may depend on the fingerprint’s ability to capture complementary structural features which are important.

#### Comparative analysis with established *in silico* tools

Toxtree achieved 100% coverage (n = 235), successfully generating predictions for all compounds in the test set, followed by SbD4Skin which achieved a coverage of 99.6%. The VEGA-based models (CAESAR and IRFMN) achieved lower coverage of 86.4% (n = 203), failing to predict approximately 14% of the dataset due to limitations in SMILES parsing or strict applicability domain constraints.

**FIGURE 10:**
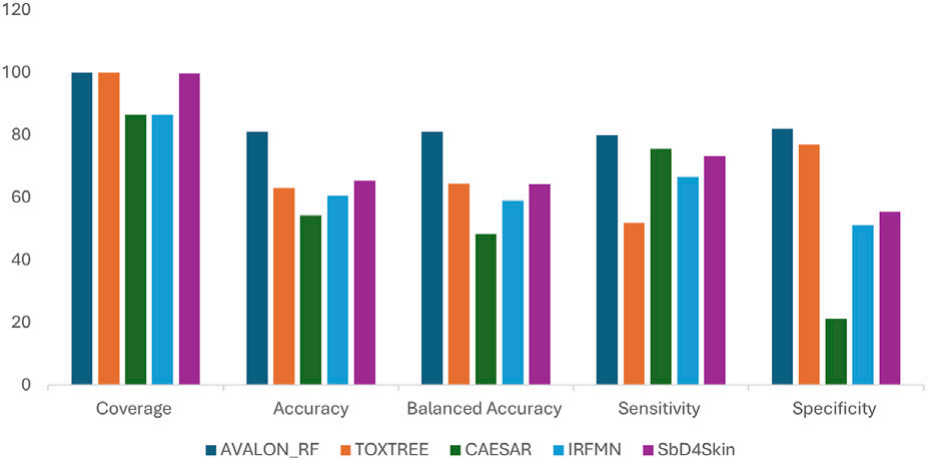
Performance comparison of the Avalon fingerprint-based Random Forest (Avalon-RF) model against four external skin sensitization prediction tools on the test set (n = 235): Toxtree, CAESAR, IRFMN, and SbD4Skin. Metrics include coverage, accuracy, balanced accuracy, sensitivity, and specificity. Avalon-RF achieved the highest overall performance (81% accuracy, 81% balanced accuracy). Among external tools, SbD4Skin showed the best accuracy (65.4%) with balanced sensitivity (73.3%) and specificity (55.3%). Toxtree favored specificity (76.9%) over sensitivity (51.9%), while CAESAR showed the reverse pattern (75.6% sensitivity, 21.2% specificity). IRFMN exhibited intermediate performance across all metrics. Coverage was 100% for Avalon-RF and Toxtree, 99.6% for SbD4Skin, and 86.4% for VEGA-based models (CAESAR and IRFMN).

Among the external tools, SbD4Skin achieved the highest Accuracy (65.4%), while Toxtree achieved the highest Balanced Accuracy (64.4%), marginally above SbD4Skin (64.3%). Toxtree exhibited a conservative prediction profile, demonstrating the highest Specificity (76.9%) among external tools, effectively minimizing false positives, but struggled with Sensitivity (51.9%), missing nearly half of the actual sensitizers. SbD4Skin showed a more balanced trade-off between Sensitivity (73.3%) and Specificity (55.3%). In contrast, CAESAR showed a strong tendency toward over-prediction. While it achieved the highest Sensitivity (75.6%) among the benchmarked tools, this came at the cost of very poor Specificity (21.2%), resulting in the lowest Balanced Accuracy (48.4%). IRFMN displayed intermediate performance with an Accuracy of 60.6% and Balanced Accuracy of 59.0%, maintaining a moderate trade-off between Sensitivity (66.7%) and Specificity (51.2%) (Figure 10).

#### Applicability domain

The applicability domain analysis demonstrated a good coverage of the test set within the chemical space of the training data. Using Avalon fingerprints (2048 bits) with a similarity threshold of S_T = 0.153 (calculated from training set parameters: $\bar{\gamma }$ = 0.106, σ = 0.094, Z = 0.5), all 235 test compounds were found to be within the applicability domain.

The distribution of average Tanimoto similarity to the k = 5 nearest neighbours for test compounds ranged from approximately 0.15–1.0, with a mean similarity of 0.521. This mean value substantially exceeds the threshold (S_T = 0.153), indicating that test compounds share considerable structural similarity with the training set.

The t-SNE visualization further confirmed the AD results, showing that test compounds are well-distributed throughout the chemical space occupied by training compounds with no apparent clustering of test compounds in regions unrepresented by training data. This spatial overlap shows that the model’s predictions for the test set are supported by structurally similar compounds in the training data, enhancing confidence in prediction reliability (Supplementary Figure S9).

## Discussion

This study demonstrates that RF significantly outperforms KNN for skin sensitization prediction, achieving 81% balanced accuracy compared to 73% for KNN when using integrated features (fingerprints, structural alerts, and physicochemical properties). Critically, RF demonstrated superior performance at handling activity cliffs. Analysis of compound clusters with ≥70% Tanimoto similarity revealed that RF predicted the correct sensitization status compared to KNN, particularly in cases where compounds shared structural alerts but exhibited different experimental outcomes. Among fingerprint approaches, substructure-based methods (Avalon, MACCS) consistently outperformed hash-based methods (Morgan, Atom Pair), with Avalon demonstrating optimal performance across metrics. SHAP interpretability analysis identified vapor pressure as the most consistently important non-structural predictor and revealed that both reactive features (Michael acceptors, Schiff base formers) and non-reactive structural elements contribute to model predictions.

Our analysis aligns with previous findings indicating that Random Forest models demonstrate optimal predictive capabilities when handling the complex, non-linear chemical spaces inherent to skin sensitization hazard assessment (Im et al., 2023; Qiao et al., 2024).

The consistent superior performance of RF across all fingerprint approaches can be attributed to its ability to handle complex, high-dimensional chemical data. KNN struggles with the “curse of dimensionality,” where high-dimensional feature spaces can lead to computational inefficiency and reduced discriminative power (Bellman, 1961; Beyer et al., 1999). This limitation was evident in KNN’s declining performance with increasingly complex feature combinations. Attempts to optimize KNN by adjusting the number of neighbors did not substantially improve performance consistent with findings by (Cosenza et al., 2021; Golden et al., 2023; Qiao et al., 2024) (Supplementary Tables 1-15). On the other hand, RF ensemble structure enables better generalization through random feature selection, reducing overfitting while maintaining accuracy (Fassnacht et al., 2014; Kongsompong et al., 2021). Additionally, this was also due to the fact that RF better handles bias and variance in integrated datasets compared to KNN, which is particularly relevant for the data integration approach used in this work (Guan and Burton, 2022).

Analysis of mispredicted compounds revealed that Toxtree exhibits both false positives (overprediction of sensitizers) and false negatives (underprediction of sensitizers) as opposed to findings by Golden et al. (2023) who reported a higher percentage of false positives than false negatives. This discrepancy can be attributed to differences in dataset composition, possible variations in stereochemistry, and distinct chemical properties of compounds in our dataset compared to previous studies.

RF exhibited more false positives, predicting non-sensitizers as sensitizers. This pattern may reflect the ensemble method’s tendency to smooth decision boundaries, potentially classifying borderline cases conservatively when uncertainty is high. The slightly imbalanced dataset (more positives than negatives) may have influenced this behavior. Conversely, KNN produced more false negatives, missing actual sensitizers. This reflects KNN’s reliance on local neighborhood patterns, which can be problematic in high-dimensional feature spaces where similar compounds may be distant due to the curse of dimensionality. KNN is particularly vulnerable in regions with overlapping classes or sparse data, leading to underestimation of sensitizers. This trend was consistent across fingerprint approaches, suggesting it may be a fundamental limitation of distance-based methods for chemical similarity assessment and might also be the case for most large-scale attempts at read-across.

Conjugated compounds were underrepresented in mispredictions (35%) for both models relative to their prevalence in the dataset (46%). This indicates that both RF and KNN models effectively captured the reactivity patterns of conjugated systems, likely due to the strong electronic signals these features provide. Similarly, cyclic compounds comprising 63% of the dataset but only 54% of mispredictions, demonstrating robust predictivity, suggesting that common structural scaffolds, such as aromatic rings and linear conjugated chains, may not be the primary drivers of mispredictions in this model.

A distinct divergence emerged in how the models handled non-conjugated species. While both models showed a bias toward missing non-conjugated sensitizers (false negatives), this trend was significantly more pronounced in RF (73% of false negatives were non-conjugated) compared to KNN (62%). This disparity may stem from the encoding specificities of the Avalon fingerprint used in the RF model. Previous studies have suggested that certain models may struggle to resolve the subtle reactivity of non-conjugated systems compared to their conjugated counterparts (Golden et al., 2023).

Notably, over half of all mispredicted compounds lacked any Toxtree structural alerts, suggesting that prediction uncertainty increases in the absence of obvious reactive motifs. However, the most striking systematic failure occurred in structurally complex compounds: those possessing multiple structural alerts (6% of the dataset). These multi-alert compounds were systematically underpredicted as false negatives by both models and exclusively so by KNN.

This systematic bias likely stems from two factors: the relative scarcity of these complex structures in the training set, and a potential ‘over-learning’ of steric deactivation. Mechanistically, the presence of multiple reactive groups can lead to steric shielding or internal reactivity that renders the molecule biologically inert in specific contexts (Golden et al., 2023). It is plausible that the models internalized this ‘shielding’ pattern, leading them to aggressively classify multi-alert compounds as non-sensitizers, thereby generating false negatives when those compounds were, in fact, active.

Our analysis indicates that the RF model outperformed KNN in predicting structurally similar compounds with discordant experimental values, particularly when these compounds shared similar structural alerts. Among the RF approaches, Avalon RF showed the best performance, highlighting its predictive advantage for skin sensitization hazard assessment. However, the evaluation of structural similarity was influenced by data availability and fingerprint selection. Atom Pair FP and Morgan FP faced limitations due to fewer pairwise relationships at the ≥70% similarity threshold, resulting in sparser chemical similarity networks. Despite these challenges, Avalon RF consistently outperformed other methods across evaluation metrics. This superior performance highlights Avalon FP’s potential utility for skin sensitization prediction. However, a significant limitation emerged during interpretability analysis: RDKit currently cannot map Avalon fingerprint bits to interpretable chemical substructures, preventing detailed SHAP feature analysis. This interpretability constraint limits regulatory applicability, as transparent, mechanistically interpretable models are preferred for toxicological decision-making. Future work should focus on developing methods to decode Avalon fingerprint features into interpretable structural elements or investigating hybrid approaches that combine Avalon’s predictive performance with the interpretability of dictionary-based fingerprints like MACCS.

Our work is also consistent with established knowledge that the choice of molecular fingerprint fundamentally dictates the representation of chemical space (Lopez Perez et al., 2025; López-Vallejo et al., 2012), which in turn significantly impacts a model’s ability to navigate complex structure-activity relationships, particularly the challenge of activity cliffs (Schuffenhauer et al., 2006; Yang et al., 2022). These results suggest that substructure-based fingerprints, particularly those that encode known reactive or toxicologically relevant motifs (e.g., PubChem, Avalon, MACCS), when coupled with a robust ensemble algorithm like Random Forest, provide an optimal framework for predicting skin sensitization. This combination proves particularly effective in resolving complex cases characterized by structural or mechanistic discordance (activity cliffs), a capability that persists even if the fingerprint does not effectively capture the chemical space. Furthermore, comparative assessment with established *in silico* tools shows that the Avalon-RF approach consistently delivers superior predictive performance.

The Avalon fingerprint, by generating smaller and more tightly knit clusters, conferred a distinct advantage to the RF model. The superior performance of the Avalon fingerprint can be attributed to its hybrid design, which combines specific substructure definitions with hashed encoding. Unlike purely hash-based methods (e.g., Morgan, Atom Pair), which produced sparse similarity networks in our analysis (Table 1), Avalon generated dense, informative clusters. This suggests that Avalon effectively captures the relevant structural signals necessary for RF classification while avoiding the ‘curse of dimensionality’ and information sparsity that hampered the hash-based approaches.

The success of Avalon FP-RF in Cluster 2 is a prime example; despite high chemical similarity and a shared Michael acceptor alert, the model correctly distinguished a non-sensitizer from three sensitizers. The tendency of PubChem FP-RF to mispredict compounds near activity cliffs suggests it struggles to resolve conflicting activity among structurally similar compounds. Similarly, the high rate of misprediction in MACCS FP-RF clusters with mechanistic discordance indicates that it may not effectively capture the subtle structural features driving differences in sensitization. While this finding is typically interpreted within the constraints of our dataset (n = 1174 with n = 235 test set), the observed clustering patterns and activity cliff performance represent specific chemical space coverage and may not generalize to all structural classes. Validation with larger, more chemically diverse datasets would strengthen these conclusions and determine whether Avalon’s advantage persists across broader applicability domains.

A clear performance hierarchy emerged between the machine learning algorithms: RF consistently outperformed KNN across all fingerprint types. The failure of Avalon FP-KNN in clusters where Avalon FP-RF succeeded underscores RF’s superior ability to model complex, non-linear relationships in structure–activity data. In contrast, KNN’s reliance on a “majority vote” from structurally similar neighbors made it vulnerable to local distortion effects. This was particularly evident in PubChem FP-KNN, where mispredictions appeared influenced by the alerts present in neighboring compounds, regardless of a compound’s true label. The broad failure of the MACCS FP-KNN combination further shows this weakness, reinforcing that KNN models are less reliable in regions with mechanistic or structural discordance, which is an important consideration for when “read-across” is data-driven as opposed to mechanistically driven.

Our similarity analysis demonstrated robust predictive performance; however, a limitation of our approach lies in the use of a 70% Tanimoto similarity cutoff. This threshold was selected because several fingerprint representations yielded an insufficient number of close analogues under more stringent similarity criteria, which would have limited meaningful activity cliff analysis. Nevertheless, a 70% cutoff remains sufficiently stringent to capture structurally related compounds while ensuring adequate sample size for evaluating model performance in activity cliff regions.

Investigating the best performing model using SHAP output, the RF model identified features more likely to be associated with lack of reactivity (e.g., aliphatic hydrocarbons) indicating that classifying a skin sensitizer based on reactive functional groups alone does not suffice. The features selected via SHAP indicate the importance of considering all molecular features, even if the model also identified chemical moieties associated with known mechanistic pathways of skin sensitization.

These findings show the informative value of the model’s predictions, capturing both reactive functional groups and physicochemical modulators such as aliphatic chains. That these features align with known sensitization mechanisms such as electrophilic reactivity and skin permeability, respectively provides some confidence that the model is leveraging chemically meaningful patterns rather than spurious correlations (Alves et al., 2018). However, further analysis revealed that the prevalence of chemical moieties in the dataset likely influences their representation and importance in model predictions. For instance, aliphatic hydrocarbons were highly prevalent in datasets, which may explain their strong contribution to prediction across fingerprint approaches. In contrast, electrophilic groups such as epoxides, sulfones, and nitriles were comparatively rare, appearing in fewer than 3% of compounds (Supplementary Figure S7). This low frequency may limit the model’s ability to reliably detect and learn from such features, particularly when using certain fingerprint types (e.g., MACCS or Atom Pair) which fail to capture the chemical space effectively. Importantly, the high prevalence of a substructure in the dataset does not necessarily imply that the model relies on it for prediction. For example, aromatic hydrocarbons, such as benzene rings, were present in nearly 50% of the dataset (Supplementary Figure S7), yet they ranked among the least important features in the SHAP analysis. This suggests that while benzene may be prevalent in the dataset and serve as a structural scaffold capable of contributing to skin sensitization when substituted with reactive moieties, its presence alone does not appear to be a strong predictor in the model’s decision-making process.

While SHAP provides a valuable interpretability layer, its ability to reveal mechanistic insights is fundamentally limited by the chemical diversity of the training data and the capacity of the molecular fingerprint to encode biologically relevant substructures. Therefore, even when SHAP identifies patterns that align with known toxicological mechanisms such as the presence of electrophiles or nucleophiles, these statistical correlations do not inherently guarantee biological insight. Meaningful interpretation requires contextualization with domain-specific knowledge to translate these patterns into robust mechanistic understanding (Cherkasov et al., 2014; Polishchuk, 2017; Sieg et al., 2019).

In addition to molecular substructures, non-structural features also carry biologically relevant information that enhanced model performance. Among these, VP emerged as a consistently informative descriptor across fingerprint approaches. Biologically, VP influences a compound’s residence time on the skin surface, a critical determinant of its ability to penetrate the stratum corneum and reach immunocompetent cells (Natsch and Gerberick, 2022). The model’s preferential use of VP over properties like LogP and WS suggests it may capture subtle differences in volatility-driven bioavailability, which is important for skin sensitization, which are often underrepresented in static *in silico* descriptors.

A limitation of this study is that physicochemical properties were primarily derived from predicted rather than experimentally measured descriptors across computational tools. While these estimates enable consistent and scalable feature generation across large chemical datasets, they may introduce uncertainty, particularly for compounds outside the applicability domain. However, this did not appear to substantially impact model performance in our analysis.

Michael acceptor alerts were consistently identified as high-importance features across all fingerprint methods, and their alignment with known electrophilic reactivity toward skin proteins supports the predictive validity of these models. This consistency across different molecular representations suggests that Michael acceptor patterns are robustly encoded and reliably captured regardless of fingerprinting approach (Enoch et al., 2011). Schiff base-forming alerts emerged as important predictive features in most models, though their contribution varied by fingerprint type. The absence of apparent Schiff base importance in Avalon and PubChem SHAP analyses may reflect either limited representation of aldehyde/ketone-amine reactive pairs in the dataset or differences in how these fingerprints encode carbonyl-amine reactivity. This fingerprint-dependent variation highlights how feature encoding can influence the apparent importance of mechanistically relevant features. Acyl transfer alerts emerged as SHAP-important features exclusively in Morgan and Atom Pair fingerprint models. This selective identification suggests these fingerprints may capture structural patterns that others miss, though whether this reflects true predictive signal or encoding artifacts warrants further investigation given the relatively uncommon occurrence of acyl transfer-mediated sensitization. SN2 alerts showed low importance across all fingerprint methods, consistent with findings by Golden et al. (2023) This likely reflects limited dataset representation rather than lack of relevance, illustrating how training data composition can constrain a model’s ability to learn certain reactive pathways.

The applicability domain analysis demonstrated that the test set compounds fall well within the chemical space of the training set, which primarily comprises diverse organic compounds. The model’s applicability domain encompasses chemically diverse compounds spanning 17 super classes, predominantly benzenoids, lipids and lipid-like molecules, organic acids and derivatives, and organoheterocyclic compounds, representing industrial and consumer chemicals. This distribution reflects the structural diversity of industrial chemicals, fragrances, preservatives, and cosmetic ingredients commonly associated with skin sensitization concerns. All test set compounds were within the AD based on Tanimoto similarity (mean = 0.521, threshold = 0.153), indicating reliable predictions across the evaluated chemical space. Compounds falling substantially outside these chemical domains should be considered outside the applicability domain, and predictions for such compounds should be interpreted with caution.

Provisional negatives were included in the dataset as a substitute to balance the proportion of negative compounds. These compounds lacked direct experimental data regarding their skin sensitization status; however, there was strong supporting evidence based on their respective CLP classifications H317 (May cause an allergic skin reaction). Within this framework, a value of 1 denotes a skin sensitizer (Category 1), which may be further sub-categorized into 1A (strong) or 1B (moderate) based on potency. Conversely, a value of 0 represents a non-classified substance. This ‘0′designation is assigned to compounds that do not meet the specific toxicological thresholds required to fulfill the criteria for sensitization (ECHA, 2025; Kättström et al., 2024). Consequently, these compounds were categorized as provisional negatives for the purpose of this study.

However, it is important to note that CLP classifications are most often applied in industrial settings, where the supporting evidence can be limited and is not always based on rigorous experimental studies. As such, while CLP values may serve as useful indicators, they should be interpreted cautiously, especially when there is no clear documentation of the experimental data used to justify the classification. From an occupational health perspective, skin sensitization remains an important endpoint, and even limited CLP-based evidence can provide meaningful context for our predictive modeling goals. Initially, we had planned to isolate these provisional negatives to assess their specific impact on model performance. However, due to the small number of such compounds in the test set (only four), statistical power for assessing their specific impact was limited. Nevertheless, descriptive analysis of these compounds’ predictions revealed they did not systematically bias model performance. In KNN models, 75% of the provisional negatives were correctly predicted as non-sensitizers across all fingerprint approaches. RF models showed similar performance, except for MACCS and Morgan fingerprints, where prediction accuracy for these compounds dropped to 50%. This fingerprint-specific variation likely reflects differences in how each method encodes structural features relevant to the characteristics of these chemicals.

While we acknowledge that including provisional negatives based on CLP classifications rather than experimental data represents a limitation, their minimal impact on overall model performance (comprising only 4 compounds in the test set, less than 2% of evaluation data) suggests this approach did not substantially compromise our conclusions. The high prediction accuracy for these compounds across most approaches further supports their provisional classification. Future studies should prioritize datasets with experimentally confirmed negative results to eliminate this uncertainty, particularly when developing models for regulatory application where data provenance is critical for acceptance.

## Conclusion

Our study emphasizes the advantages of the RF model compared to KNN for skin sensitization hazard assessment. RF demonstrably utilizes the information within training data more effectively than KNN, leading to better predictive performance. Additionally, RF consistent improvement across different datasets suggests it is less prone to overfitting. However, there is room for further improvement. Incorporating features derived from *in vitro* assays, such as KeratinoSens™, direct peptide reactivity tests, and h-CLAT, could potentially enhance RF performance. Notably, the model appears adept at handling the addition of new features, suggesting a promising avenue for improvement. For KNN, addressing its limitations might involve implementing well-established machine learning techniques like feature selection using SHAP. These techniques can streamline the training data, ensuring KNN focuses on the most critical features for better prediction. The SHAP analysis method employed in this study offers a valuable resource for developers of computational toxicology tools. By understanding how features contribute to model predictions, developers can refine their data and models for better performance. Furthermore, the study emphasizes the importance of evaluating model performance based on chemical fingerprints and similarity particularly for QSAR based models. This approach can lead to more robust read-across. Its benefit extends beyond skin sensitization as it can be applied across other toxicological domains. Building upon these findings, future research could explore transformer or graph-based deep learning approaches that allow for better representation techniques such as positional encoding with 2D or 3D chemical data representation for skin sensitization prediction with even more complex datasets. Transfer learning also holds significant promise for skin sensitization prediction, as it enables broader integration of chemical space via encoders pre-trained on large-scale chemical datasets. More importantly, these models are well-suited to handling diverse data types, making them particularly valuable for integrative approaches. This adaptability supports the prediction of sensitization profiles using both binary and multiclass classification frameworks, thereby enhancing their generalizability and applicability.

## Data Availability

The original contributions presented in the study are included in the article/Supplementary Material, further inquiries can be directed to the corresponding author.
