# Supplementary material for: Optimizing skin sensitization prediction across activity cliffs: a comparative analysis of K-nearest neighbours vs random Forest
Source: Front Toxicol. 2026 Aug 3;8:1739746. doi: 10.3389/ftox.2026.1739746 (PMC13477787; doi:10.3389/ftox.2026.1739746)
Supplement: Supplementary file 1 [file DataSheet1.docx]

**OPTIMIZING SKIN SENSITIZATION PREDICTION: A COMPARATIVE ANALYSIS OF KNN VS RANDOM FOREST**

**Daniel C. Ukaegbu^1^, Karolina Kopańska^1^, Peter Ranslow^2^, Alexandra Maertens^1*^**

^1^ Center for Alternatives to Animal Testing (CAAT), Johns Hopkins Bloomberg School of Public Health, Baltimore, Maryland 21205, United States

^2^ Consortium for Environmental Risk Management (CERM), Hallowell, Maine 04347, United States

*Correspondence:

Alexandra Maertens

amaerte1@jhu.edu

**Supplementary Figure 1.** Cross-validation of physicochemical property predictions between computational tools. (A) Water solubility (WS) predictions from OPERA versus CHEMBCPP (n=660; Pearson r=0.929, RMSE=0.599, MAE=0.378). (B) Vapor pressure (VP) predictions from OPERA versus CHEMBCPP (n=660; Pearson r=0.937, RMSE=0.860, MAE=0.577). (C) LogP predictions from OPERA versus DataWarrior (n=669; Pearson r=0.933, RMSE=0.789, MAE=0.517). Regression lines (red), 1:1 identity lines (dashed black), and 95% confidence intervals (shaded) are shown. Strong correlations across all properties support the interchangeable use of these tools for gap-filling missing values.

**Supplementary Figure 2**: Supplementary Figure 1. Workflow for curation of the skin sensitization dataset. Data were collected from e-Chemportal (2,626 chemicals), curated with ECHA dossiers (827 sensitizers, 164 non-sensitizers), augmented with negatives from Golden et al. (2021) and the USEPA HPVC database, and finalized after excluding metals/metals salts. The resulting dataset comprised 1,174 chemicals (656 sensitizers, 518 non-sensitizers).

**MODEL PERFORMANCE WITH STRUCTURAL FINGERPRINTS ALONE**

**Table 1- 5** summarizes the performance of the fingerprint(only)-based approach across varying K-values (K-nearest neighbors) and all evaluation metrics across test set(n=235). While increasing the K-value may suggest improved performance, the results demonstrate that this can lead to overfitting and poor generalization,

**PUBCHEM FP**

**Supplementary Table 1:**

| **Metrics** | **KNN3** | **KNN4** | **KNN5** | **KNN6** | **KNN7** |
| --- | --- | --- | --- | --- | --- |
| **Accuracy** | 71 | 72 | 72 | 71 | 70 |
| **Precision** | 78 | 78 | 80 | 79 | 78 |
| **Recall** | 67 | 69 | 66 | 64 | 64 |
| **F1-score** | 72 | 73 | 72 | 71 | 70 |

**AVALON FP**

**Supplementary Table 2:**

| **Metrics** | **KNN3** | **KNN4** | **KNN5** | **KNN6** | **KNN7** |
| --- | --- | --- | --- | --- | --- |
| **Accuracy** | 71 | 74 | 68 | 72 | 67 |
| **Precision** | 80 | 81 | 79 | 81 | 78 |
| **Recall** | 68 | 68 | 60 | 66 | 57 |
| **F1-score** | 74 | 74 | 68 | 73 | 66 |

**MACCS FP**

**Supplementary Table 3:**

| **Metrics** | **KNN3** | **KNN4** | **KNN5** | **KNN6** | **KNN7** |
| --- | --- | --- | --- | --- | --- |
| **Accuracy** | 68 | 68 | 65 | 66 | 67 |
| **Precision** | 77 | 77 | 73 | 74 | 77 |
| **Recall** | 62 | 62 | 58 | 58 | 58 |
| **F1-score** | 69 | 68 | 64 | 65 | 66 |

**ATOM PAIR FP**

**Supplementary Table 4:**

| **Metrics** | **KNN3** | **KNN4** | **KNN5** | **KNN6** | **KNN7** |
| --- | --- | --- | --- | --- | --- |
| **Accuracy** | 64 | 66 | 65 | 66 | 63 |
| **Precision** | 75 | 77 | 77 | 80 | 76 |
| **Recall** | 53 | 56 | 53 | 52 | 49 |
| **F1-score** | 62 | 65 | 62 | 63 | 60 |

**MORGAN FP**

**Supplementary Table 5:**

| **Metrics** | **KNN3** | **KNN4** | **KNN5** | **KNN6** | **KNN7** |
| --- | --- | --- | --- | --- | --- |
| **Accuracy** | 65 | 63 | 63 | 61 | 60 |
| **Precision** | 77 | 76 | 77 | 76 | 81 |
| **Recall** | 52 | 49 | 47 | 43 | 36 |
| **F1-score** | 62 | 59 | 58 | 55 | 50 |

**STRUCTURAL FINGERPRINT AND STRUCTURAL ALERT**

**Figure 6-10**: The results here indicate that increasing data complexity (intended to enhance model performance) does not consistently translate to improvements in generalization as performance across the different evaluation metrics tend not to improve as K-nearest neighbor was increased

**PUBCHEM FP**

**Supplementary Table 6:**

| **Metrics** | **KNN3** | **KNN4** | **KNN5** | **KNN6** | **KNN7** |
| --- | --- | --- | --- | --- | --- |
| **Accuracy** | 72 | 71 | 72 | 74 | 71 |
| **Precision** | 79 | 80 | 80 | 81 | 79 |
| **Recall** | 68 | 66 | 67 | 69 | 64 |
| **F1-score** | 73 | 72 | 73 | 74 | 71 |

**AVALON FP**

**Supplementary Table 7:**

| **Metrics** | **KNN3** | **KNN4** | **KNN5** | **KNN6** | **KNN7** |
| --- | --- | --- | --- | --- | --- |
| **Accuracy** | 72 | 74 | 69 | 72 | 68 |
| **Precision** | 81 | 82 | 80 | 80 | 80 |
| **Recall** | 65 | 67 | 59 | 67 | 58 |
| **F1-score** | 72 | 74 | 68 | 72 | 67 |

**MACCS FP**

**Supplementary Table 8:**

| **Metrics** | **KNN3** | **KNN4** | **KNN5** | **KNN6** | **KNN7** |
| --- | --- | --- | --- | --- | --- |
| **Accuracy** | 70 | 70 | 66 | 69 | 67 |
| **Precision** | 78 | 78 | 75 | 79 | 79 |
| **Recall** | 64 | 64 | 58 | 60 | 56 |
| **F1-score** | 70 | 70 | 65 | 68 | 65 |

**ATOM PAIR FP**

**Supplementary Table 9:**

| **Metrics** | **KNN3** | **KNN4** | **KNN5** | **KNN6** | **KNN7** |
| --- | --- | --- | --- | --- | --- |
| **Accuracy** | 63 | 67 | 64 | 67 | 63 |
| **Precision** | 75 | 79 | 78 | 81 | 76 |
| **Recall** | 52 | 56 | 50 | 52 | 50 |
| **F1-score** | 61 | 65 | 61 | 64 | 60 |

**MORGAN FP**

**Supplementary Table 10:**

| **Metrics** | **KNN3** | **KNN4** | **KNN5** | **KNN6** | **KNN7** |
| --- | --- | --- | --- | --- | --- |
| **Accuracy** | 66 | 64 | 62 | 63 | 60 |
| **Precision** | 88 | 78 | 80 | 80 | 82 |
| **Recall** | 51 | 49 | 43 | 43 | 36 |
| **F1-score** | 63 | 60 | 55 | 56 | 50 |

**STRUCTURAL FINGERPRINT, STRUCTURAL ALERT AND PSYCHEM PROPERTIES**

**Figure 11-16:** Growing data complexity and increased data dimensionality aimed at improving performance does deteriorate performance. Results from this domain validate this observation as performance across the different evaluation metrics tend not to improve as expected as K-nearest neighbor was increased.

**PUBCHEM FP**

**Supplementary Table 11:**

| **Metrics** | **KNN3** | **KNN4** | **KNN5** | **KNN6** | **KNN7** |
| --- | --- | --- | --- | --- | --- |
| **Accuracy** | 71 | 71 | 72 | 72 | 71 |
| **Precision** | 79 | 79 | 80 | 79 | 79 |
| **Recall** | 66 | 67 | 66 | 67 | 65 |
| **F1-score** | 72 | 72 | 73 | 72 | 72 |

**AVALON FP**

**Supplementary Table 12:**

| **Metrics** | **KNN3** | **KNN4** | **KNN5** | **KNN6** | **KNN7** |
| --- | --- | --- | --- | --- | --- |
| **Accuracy** | 71 | 73 | 69 | 72 | 68 |
| **Precision** | 79 | 81 | 80 | 80 | 80 |
| **Recall** | 67 | 67 | 60 | 67 | 58 |
| **F1-score** | 72 | 73 | 68 | 73 | 67 |

**MACCS FP**

**Supplementary Table 13:**

| **Metrics** | **KNN3** | **KNN4** | **KNN5** | **KNN6** | **KNN7** |
| --- | --- | --- | --- | --- | --- |
| **Accuracy** | 68 | 66 | 67 | 69 | 66 |
| **Precision** | 78 | 75 | 78 | 79 | 77 |
| **Recall** | 60 | 59 | 59 | 61 | 56 |
| **F1-score** | 68 | 66 | 66 | 69 | 65 |

**MORGAN FP**

**Supplementary Table 14:**

| **Metrics** | **KNN3** | **KNN4** | **KNN5** | **KNN6** | **KNN7** |
| --- | --- | --- | --- | --- | --- |
| **Accuracy** | 64 | 66 | 64 | 66 | 63 |
| **Precision** | 75 | 78 | 78 | 81 | 76 |
| **Recall** | 52 | 55 | 50 | 52 | 49 |
| **F1-score** | 62 | 65 | 61 | 64 | 60 |

**ATOM PAIR FP**

**Supplementary Table 15:**

| **Metrics** | **KNN3** | **KNN4** | **KNN5** | **KNN6** | **KNN7** |
| --- | --- | --- | --- | --- | --- |
| **Accuracy** | 66 | 66 | 63 | 64 | 62 |
| **Precision** | 83 | 80 | 80 | 83 | 89 |
| **Recall** | 51 | 53 | 45 | 45 | 37 |
| **F1-score** | 63 | 64 | 58 | 58 | 52 |

| **Fingerprints** | **Accuracy (%)** | **Precision (%)** | **Recall (%)** | **F1-Score (%)** |
| --- | --- | --- | --- | --- |
| **MACCS FP** | 68 | 69 | 69 | 68 |
| **AVALON FP** | 67 | 68 | 68 | 67 |
| **MORGAN FP** | 59 | 62 | 61 | 58 |
| **PUBCHEM FP** | 67 | 69 | 69 | 68 |
| **ATOM PAIR FP** | 64 | 66 | 66 | 64 |
| **Supplementary Table 16**. Summary of KNN Model after 10-fold cross validation performance with fingerprints and structural alert and physiochemical properties on train dataset (n= 939). | | | | |

| **Fingerprints** | **Accuracy (%)** | **Precision (%)** | **Recall (%)** | **F1-Score (%)** |
| --- | --- | --- | --- | --- |
| **MACCS FP** | 73 | 72 | 72 | 72 |
| **AVALON FP** | 74 | 74 | 73 | 73 |
| **MORGAN FP** | 70 | 70 | 69 | 69 |
| **PUBCHEM FP** | 72 | 72 | 71 | 71 |
| **ATOM PAIR FP** | 72 | 71 | 71 | 71 |
| **Supplementary Table 17** . Random Forest model performance using 10-fold cross-validation on the train dataset (n= 939) with integrated features (fingerprints, structural alerts, and physicochemical properties | | | | |

**Table 18:** Performance Metrics for Random Forest Models using Molecular Fingerprints on test set (n=235). > Note: ACC = Accuracy; PRE = Precision; REC = Recall; F1 = F1-Score; SPE = Specificity; SEN = Sensitivity; BA = Balanced Accuracy. All values are expressed as percentages (%).

| **Fingerprints** | **ACC (%)** | **PRE (%)** | **REC (%)** | **F1 (%)** | **SPE (%)** | **SEN (%)** | **BA (%)** |
| --- | --- | --- | --- | --- | --- | --- | --- |
| MACCS FP | 73 | 75 | 78 | 77 | 75 | 70 | 73 |
| AVALON FP | 77 | 78 | 83 | 80 | 78 | 77 | 78 |
| MORGAN FP | 71 | 75 | 72 | 74 | 75 | 67 | 71 |
| PUBCHE FP | 73 | 74 | 78 | 76 | 75 | 71 | 73 |
| RDKIT FP | 73 | 75 | 77 | 76 | 75 | 67 | 71 |

**Table 19:** Summary of Random Forest model performance with fingerprints integrated with structural alert (SNAR, Schiff base, Michael acceptor, SNAR, SN2)

| **Fingerprints** | **ACC (%)** | **PRE (%)** | **REC (%)** | **F1 (%)** | **SPE (%)** | **SEN (%)** | **BA (%)** |
| --- | --- | --- | --- | --- | --- | --- | --- |
| MACCS FP | 75 | 76 | 81 | 78 | 76 | 74 | 75 |
| AVALON FP | 79 | 79 | 85 | 82 | 79 | 79 | 79 |
| MORGAN FP | 71 | 74 | 73 | 73 | 74 | 67 | 71 |
| PUBCHE FP | 75 | 77 | 79 | 78 | 77 | 73 | 75 |
| RDKIT FP | 70 | 73 | 74 | 73 | 73 | 67 | 70 |

**Table 20**: Summary of Random Forest model performance with fingerprints integrated structural alert and physiochemical properties ( LogP, VP, W.SOL)

| **Fingerprints** | **ACC (%)** | **PRE (%)** | **REC (%)** | **F1 (%)** | **SPE (%)** | **SEN (%)** | **BA (%)** |
| --- | --- | --- | --- | --- | --- | --- | --- |
| MACCS FP | 74 | 74 | 82 | 78 | 74 | 74 | 74 |
| AVALON FP | 81 | 82 | 85 | 83 | 82 | 80 | 81 |
| MORGAN FP | 73 | 74 | 79 | 77 | 75 | 72 | 74 |
| PUBCHE FP | 77 | 77 | 83 | 80 | **77** | **76** | **77** |
| RDKIT FP | 73 | 74 | 80 | 77 | 74 | 73 | 74 |

**Supplementary Figure 3**. Confusion matrix components for skin sensitization prediction models across five molecular fingerprint types(n=235). (A) K-Nearest Neighbors (KNN) and (B) Random Forest (RF) classifiers showing true positives (TP), true negatives (TN), false positives (FP), and false negatives (FN) for MACCS, Avalon, Morgan, PubChem, and Atom Pair fingerprints. Higher TP and TN values indicate better predictive performance, while lower FP and FN values reflect reduced misclassification rates.

**Supplementary Figure 4.** Misprediction analysis for K-Nearest Neighbors (KNN) skin sensitization models across five molecular fingerprint types (MACCS, Avalon, Morgan, PubChem, and Atom Pair). Compounds are grouped by structural features: conjugation status (conjugated vs. non-conjugated), ring presence (has ring vs. no ring), ring count (1 ring vs. >1 ring), and structural alert count (0, 1, or >1 alerts). (A) Total mispredictions (FP + FN), (B) false positives (FP), and (C) false negatives (FN) are shown for each fingerprint and structural category.

**Supplementary Figure 5.** Misprediction analysis for Random Forest (RF) skin sensitization models across five molecular fingerprint types (MACCS, Avalon, Morgan, PubChem, and Atom Pair). Compounds are grouped by structural features: conjugation status (conjugated vs. non-conjugated), ring presence (has ring vs. no ring), ring count (1 ring vs. >1 ring), and structural alert count (0, 1, or >1 alerts). (**A**) Total mispredictions (FP + FN), (**B**) false positives (FP), and (**C**) false negatives (FN) are shown for each fingerprint and structural category.

**Supplementary Figure 6: SHAP feature importance analysis for Avalon RF**

Horizontal bar plots show the mean SHAP values (average impact on model output) for the top molecular and non-molecular features. Blue bars represent contributions toward Class 0 (non-sensitizers), red bars represent contributions toward Class 1 (sensitizers). Features are ranked by total importance magnitude. features.

**Supplementary Figure 7**. This figure shows the prevalence of selected molecular substructures across the dataset. Benzene rings and tertiary amines were the most common, appearing in approximately 41% and 24% of the dataset, respectively. Other frequently occurring moieties included halogens, primary and secondary amines, hydroxyl groups, and phenols. Less common substructures such as nitriles, epoxides, sulfones, and aromatic moieties were present in fewer than 5% of compounds. These distributions reflect the chemical diversity of the dataset and highlight which reactive or functional groups are most represented in the compounds analyzed.

**Supplementary Figure 8:** Boxplot showing the variability in physicochemical properties (LogP, VP, and WS) in the dataset. The distribution of LogP and WS shows a moderate spread around the median with multiple outliers, while VP exhibits the highest variability, as indicated by its larger interquartile range and whiskers extending further from the median.

**Supplementary Figure 9:** Applicability domain assessment for the test set using Avalon fingerprints (k=5, Z=0.5). (A) Distribution of average Tanimoto similarity to the five nearest neighbors in the training set. All test compounds (n=235) exceeded the similarity threshold (S_T=0.153, dashed line), with a mean similarity of 0.521 (dotted line). Training set parameters: γ̄=0.106, σ=0.094. (B) t-SNE visualization of chemical space showing training set compounds (gray circles) and test set compounds (green stars). Test compounds are well-distributed throughout the training chemical space, confirming all compounds fall within the applicability domain.

**MACCS FP**

| **FP INDEX** | **SUBSTRUCTURE** |
| --- | --- |
| 134_MF | Halogen |
| 88_MF | Sulfur |
| 132_MF | OACH2A |
| 156_MF | NA(A)A |
| 142_MF | N > 1 |
| 138_MF | QCH2A>1A: |
| 118_MF | ACH2CH2A > 1 |
| 99_MF | C=C |
| 161_MF | N |
| 115_MF | CH3ACH2A |
| 158_MF | C-N |
| 146_MF | O > 2 |
| 34_MF | CH2=A |
| 114_MF | CH3CH2A |
| **Supplementary Table 21**. MACCS fingerprint bit indices and corresponding substructures generated by RDKit using 166 predefined SMARTS patterns. Substructure definitions obtained from GitHub repository dictionary for MACCS fingerprints. (https://github.com/rdkit/rdkit/blob/master/rdkit/Chem/MACCSkeys.py) | |

**MORGAN FP**

| **FP INDEX** | **RDKIT SUBSTRUCTURE** |
| --- | --- |
| 19_MF |  |
| 80_MF |  |
| 61_MF |  |
| 4_MF |  |
| 6_MF |  |
| 131_MF |  |
| 8_MF |  |
| 27_MF |  |
| 280_MF |  |
| 187_MF |  |
| 464_MF |  |
| 417_MF |  |
| 4_MF |  |
| 71_MF |  |
| 122_MF |  |
| 6_MF |  |
| 474_MF |  |
| **Supplementary Table 22**. Morgan fingerprint bit indices and corresponding substructures generated by RDKit. Morgan fingerprints use circular topology that identifies atoms within a specified radius of each central atom and maps structural environments to integer hash codes (FP_INDEX) | |

**ATOM PAIR**

| 248_MF |  |
| --- | --- |
| 484_MF |  |
| 57_MF |  |
| 412_MF |  |
| 160_MF |  |
| 240_MF |  |
| 17_MF |  |
| 189_MF |  |
| 153_MF |  |
| 280_MF |  |
| 188_MF |  |
| 497_MF |  |
| 164_MF |  |
| 372_MF |  |
| 433_MF |  |
| **Supplementary Table 23**. Atom pair fingerprint bit indices and corresponding substructures generated by RDKit atom pair fingerprint generator. This method enumerates heavy atom pairs at various topological distances and hashes them into a fixed-length bitstring. | |

**PUBCHEM FP**

| FINGERPRINT INDEX | SMART SUBSTRUCTURE |
| --- | --- |
| 15_MF | >=2N |
| 14_MF | >=1N |
| 33_MF | >=1S |
| 37_MF | >= 1 Cl |
| 185_MF | >= 2 any ring size 6 |
| 285_MF | C-N |
| 293_MF | C-S |
| 294_MF | C-Cl |
| 333_MF | C(~C)(~C)(~C) |
| 351_MF | C(~C)(~N) |
| 390_MF | N(~C)(~C) |
| 416_MF | C=C |
| 464_MF | N-C-C=C |
| 540_MF | C-N-C-[#1] |
| 553_MF | O=C-C=C |
| 672_MF | O=C-C=C-[#1] |
| **Supplementary Table 24**. PubChem fingerprint bit indices and corresponding substructures. PubChem fingerprints use a dictionary-based approach incorporating 881 structural features organized into seven categorical sections. https://www.pingzhang.net/bak/drugreposition/list_fingerprints.pdf | |
